# Total synthesis of decarboxyaltenusin

**DOI:** 10.3762/bjoc.17.22

**Published:** 2021-01-22

**Authors:** Lucas Warmuth, Aaron Weiß, Marco Reinhardt, Anna Meschkov, Ute Schepers, Joachim Podlech

**Affiliations:** 1 Institute of Organic Chemistry, Karlsruhe Institute of Technology (KIT), 76131 Karlsruhe, Fritz-Haber-Weg 6, Germany; 2 Institute of Toxicology and Genetics, Karlsruhe Institute of Technology (KIT), 76344 Eggenstein-Leopoldshafen, Hermann-von-Helmholtz-Platz 1, Germany

**Keywords:** biaryls, boronates, mycotoxins, polyketides, Suzuki coupling

## Abstract

The total synthesis of decarboxyaltenusin (5’-methoxy-6-methyl-[1,1’-biphenyl]-3,3’,4-triol), a toxin produced by various mold fungi, has been achieved in seven steps in a yield of 31% starting from 4-methylcatechol and 1-bromo-3,5-dimethoxybenzene, where the longest linear sequence consists of five steps. The key reaction was a palladium-catalyzed Suzuki coupling of an aromatic boronate with a brominated resorcin derivative.

## Introduction

**Scheme 1:**
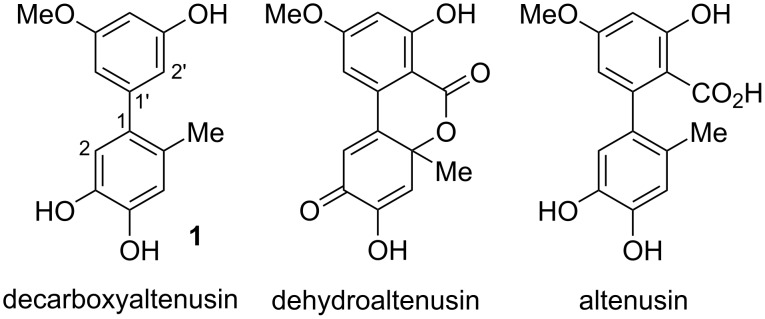
Biphenyl-derived mycotoxins.

5’-Methoxy-6-methyl-[1,1’-biphenyl]-3,3’,4-triol (**1**, Scheme 1) has been first mentioned 1974 as a reduction and decarboxylation product of dehydroaltenusin [1]. As the compound has later been isolated from *Ulocladium* sp. [2], *Nigrospora sphaerica*, *Phialophora* sp. [3], *Penicillium pinophilum* SD-272 [4], *Alternaria* sp. [3,5–6], and from the endophytic fungus *Botryosphaeria dothidea* in *Melia azedarach* [7] it turned out to be a widely occurring natural product. It shows marked DPPH radical scavenging activities with determined IC_50_ values of 18.7 ± 0.2 μM [7] and 148 ± 3 μM [2], respectively, and displays inhibitory activity against three tyrosine kinases (EGFR, VEGFR-1, and c-Met) [5]. As mentioned, it is accessible through the reduction of dehydroaltenusin with zinc powder in acetic acid with intermediate formation of altenusin [1]. Nevertheless, this route cannot be considered as a viable approach to this compound due to the reduced accessibility of the precursors and since no experimental details have been published for the transformation. To continue our efforts in the total synthesis of mycotoxins [8–18] and to provide larger amounts of the polyketide **1** sufficient for thorough biological investigations (as have been suggested by the European Food Safety Authority, EFSA [19]), we considered it useful to supply a more straightforward synthesis of this compound, for which we here propose the obvious name decarboxyaltenusin.

## Results and Discussion

**Scheme 2:**
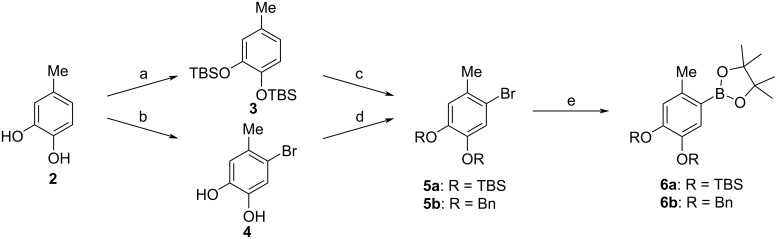
Synthesis of arylboronates **6**. Conditions: a) TBSCl, DMAP, imidazole, DMF, 50 °C, 4 h (96%); b) NBS, MeCN, rt, 71 h (quant.); c) NBS, MeCN, rt, 72 h (R = TBS, 96%); d) BnBr, KI, K_2_CO_3_, DMF/acetone, 70 °C, 29 h, (R = Bn, 86%); e) R = TBS: BuLi, 2-isopropoxy-4,4,5,5-tetramethyl-1,3,2-dioxaborolane, THF, −78 °C, 0.45–2 h, rt, 18 h (57%); R = Bn: bis(pinacolato)diboron, Pd(dppf)Cl_2_·CH_2_Cl_2_ (cat.), KOAc, dioxane, 80 °C, 17 h (55%).

In a retrosynthetic analysis, we envisioned a Suzuki coupling of two suitably substituted arenes. Silyl protecting groups like the *tert*-butyldimethylsilyl group (TBS) were considered appropriate for all projected reaction steps. The boronate moiety **6a** was prepared starting with 4-methylcatechol (**2**), which was initially protected with *tert*-butyldimethylsilyl chloride in the presence of 4-(dimethylamino)pyridine (DMAP) and imidazole (Scheme 2) according to a published procedure [20]. The thus obtained bis(silylether) **3** was then brominated with *N*-bromosuccinimide (NBS), where the utilization of acetonitrile as solvent [21] instead of carbon tetrachloride [22] furnished a close to quantitative yield of bromide **5a**, though a prolonged reaction time of 72 h had to be accepted. The subsequent formation of boronate **6a** was accomplished through a metal–halogen interchange with butyllithium and trapping with 2-isopropoxy-4,4,5,5-tetramethyl-1,3,2-dioxaborolane [23].

**Scheme 3:**
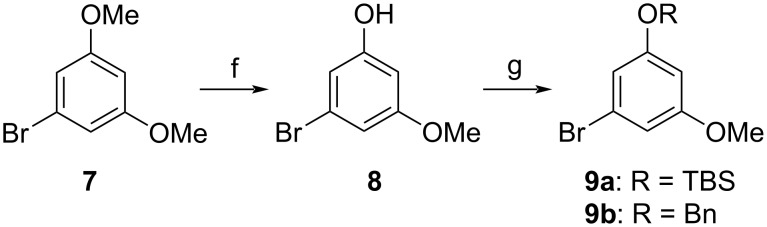
Synthesis of aryl bromides **9**. Conditions: f) BBr_3_, −78 °C to rt, 18 h (71%); g) R = TBS: TBSCl, DMAP, imidazole, DMF, 55 °C, 4 h (73%); R = Bn: BnBr, K_2_CO_3_, DMF/acetone 1:2, 80 °C, 43 h (98%).

The electrophilic compound suitable for the projected cross coupling was obtained by mono-demethylation of commercially available 1-bromo-3,5-dimethoxybenzene (**7**) with boron tribromide (Scheme 3). A satisfactory yield of phenol **8** was observed with 0.9 equivalents of the Lewis acid, while the utilization of 1.5 equivalents led to a significant overreaction with the predominant formation of 5-bromobenzene-1,3-diol (59%) together with a smaller amount of the required product **8** (30%). The subsequent protection with the TBS group yielded the known aryl bromide **9a** [24] with a 73% yield.

**Scheme 4:**
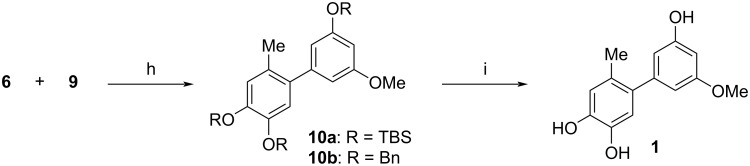
Final steps in the synthesis of biaryl **1**. Conditions: h) Pd(OAc)_2_, SPhos, Cs_2_CO_3_, dioxane/H_2_O 7:1, 70 °C, 18 h, (R = TBS: 98%, containing non-separable impurities; R = Bn: 89%) ; i) R = Bn: Pd/C (10%), H_2_, THF, 8 bar, 24 h, 40 °C (88%).

The Suzuki coupling of boronate **6a** and aryl bromide **9a** using palladium acetate and cesium carbonate in the presence of the ligand SPhos [25] yielded biaryl **10a** with virtually quantitative yield (98%, Scheme 4). Unfortunately, the removal of non-identified byproducts and of **9a** (which had been used in a 1.2-fold excess) turned out to be very laborious. Moreover, the subsequent deprotection to the natural product **1** could not be achieved sufficiently: After treatment of **10a** with tetrabutylammonium fluoride (TBAF), the signals of **1** could be detected in a ^1^H NMR spectrum of the crude product, but purification and isolation of the product by column chromatography was not possible ‒ neither with conventional nor with reversed phase methods. It turned out that the high polarity of the triol complicated its separation from other polar side products.

To circumvent this problem, we decided to use a different protection group strategy and to employ hydrogenolytically cleavable benzyl groups. The synthesis of the benzyl-protected boronate was here achieved with a modified strategy including bromination [21] of 4-methylcatechol (**2**) to the known bromide **4** [26] and subsequent benzyl protection to the bis(benzyl ether) **5b** using standard conditions (Scheme 2) [27]. The preparation of boronate **6b** applying the conditions used for the silylated substrate **6a** (vide supra) led to a mediocre 44% yield, but the utilization of a palladium-catalyzed borylation with bis(pinacolato)diboron afforded the product with 55% yield. The *O*-benzylation of phenol **8** furnishing the bromide **9b** was accomplished with virtually quantitative yield. Suzuki coupling of the benzyl-protected compounds **6b** and **9b** led to biaryl **10b** with 89% yield; it was deprotected with palladium on charcoal under eight bar hydrogen pressure (Scheme 4). The product **1** could now be purified by a simple chromatography on silica gel and was obtained in 88% yield.

NMR spectroscopic data of the natural product had been published by Wang et al. [2] and by Xiao et al. [7] (Table 1). A comparison of the ^13^C NMR data of the now synthesized compound with Wang’s data shows a systematic deviation of about −0.5 ppm (possibly due to a calibration inaccuracy in the original paper) and a deviant signal around 144 ppm, which is a further −0.4 ppm off (marked in boldface). Nevertheless, all signals published by Xiao et al. are in virtually perfect agreement with the NMR data measured by us, what leaves no reasonable doubt that the synthesized structure **1** is identical with the natural product.

**Table 1:** NMR data of natural and synthetic biaryl **1**.

natural product		synthetic product		deviation^a^
δ [ppm]^b,c^	δ [ppm]^c,d^	signal	δ [ppm]	Δδ [ppm]

19.8	19.50	6-Me	19.4	−0.4/−0.1
55.4	54.97	OMe	54.9	−0.5/−0.1
99.7	99.21	C-4’	99.2	−0.5/±0.0
106.2	105.80	C-2’	105.8	−0.4/±0.0
109.3	108.84	C-6’	108.8	−0.5/±0.0
117.1	116.70	C-2	116.7	−0.4/±0.0
118.0	117.56	C-5	117.5	−0.5/−0.1
125.3	124.89	C-6	124.8	−0.5/−0.1
132.7	132.30	C-1	132.3	−0.4/±0.0
143.5^e,f^	142.92	C-1’	142.9	−0.6/±0.0
**144.5**^e,f^	143.66	C-3	**143.6**	**−0.9**/−0.1
144.8^e,f^	144.39	C-4	144.3	−0.5/−0.1
158.5	158.06	C-3’	158.0	−0.5/−0.1
160.4	160.04	C-5’	160.0	−0.4/±0.0
2.05 (s)		6-CH_3_	2.05 (s)	±0.00
3.69 (s)		5’-OCH_3_	3.70 (s)	+0.01
6.20 (br. s)		6’-H	6.20 (dd)	±0.00
6.21 (br. s)		2’-H	6.22 (t)	+0.01
6.25 (br. s)		4’-H	6.25 (t)	±0.00
6.54 (s)		2-H	6.55 (s)	+0.01
6.60 (s)		5-H	6.60 (s)	±0.00
8.72 (br. s)		3-OH	8.72 (br. s)	±0.00
8.78 (br. s)		4-OH	8.78 (br. s)	±0.00
9.42 (br. s)		3’-OH	9.37 (br. s)	− 0.05

^a^Deviation of the synthesized product’s data from published data: Wang et al. [2]/Xiao et al. [7]; ^b^data published by Wang et al. [2]; ^c^the data are given in ascending order. Assignments in the original papers are in agreement with those given for the synthesized product (except footnote e); ^d^data published by Xiao et al. [7]; ^e^the assignment in the original paper is: 143.5: C-4, 144.5: C-3, and 144.8: C-1’; ^f^a superscript letter is included after these numbers in the original paper, but a corresponding footnote is missing. It can be assumed that the assignment of these signals had been considered questionable.

Decarboxyaltenusin (**1**) was screened for toxicity towards human HeLa cells but proved nontoxic at biologically relevant concentrations and showed an LD_50_ value of above 50 μM. This screening was performed by measuring the cell viability using an MTT assay, where the viability is assessed based upon the reduction of the yellow tetrazolium MTT [3-(4,5-dimethylthiazolyl-2)-2,5-diphenyltetrazolium bromide] by metabolically active and hence viable cells. The resulting intracellular purple formazan was quantified spectrophotometrically. The cell viability was calculated based on an untreated control. The cell viability cut-off was <70%. At a 0.5 μM concentration of decarboxyaltenusin (**1**), it was 103% ± 1%, at 5 μM it was 94% ± 1.4%, and at 50 μM 92% ± 1%.

## Conclusion

The total synthesis of decarboxyaltenusin (**1**) was achieved in seven steps in a yield of 31% starting from 4-methylcatechol (**2**) and 1-bromo-3,5-dimethoxybenzene (**7**), where the longest linear sequence has consisted of five steps. Decarboxyaltenusin turned out to be nontoxic towards human HeLa cells.

## Supporting Information

File 1 Experimental procedures and NMR spectra of all new compounds and of decarboxyaltenusin (**1**).
