# Supplementary material for: Total synthesis of decarboxyaltenusin
Source: Beilstein J Org Chem. 2021 Jan 22;17:224–8. doi: 10.3762/bjoc.17.22 (PMC7849237; doi:10.3762/bjoc.17.22)

BEILSTEIN JOURNAL OF ORGANIC CHEMISTRY

# Supporting Information

for

# Total synthesis of decarboxyaltenusin

Lucas Warmuth, Aaron Weiß, Marco Reinhardt, Anna Meschkov, Ute Schepers and Joachim Podlech

*Beilstein J. Org. Chem.* **2021,** *17,* 224–228. doi:10.3762/bjoc.17.22

# Experimental procedures and NMR spectra of all new compounds and of decarboxyaltenusin (1)

License and Terms: This is a supporting information file under the terms of the Creative Commons Attribution License (https://creativecommons.org/licenses/by/4.0). Please note that the reuse, redistribution and reproduction in particular requires that the author(s) and source are credited and that individual graphics may be subject to special legal provisions.
The license is subject to the *Beilstein Journal of Organic Chemistry* terms and conditions: (https://www.beilstein-journals.org/bjoc/terms)

# Table of contents


## A. Experimental procedures

General

1,2-Bis(*tert*-butyldimethylsilyloxy)-4-methylbenzene (**3**)

1-Bromo-3,4-bis(*tert*-butyldimethylsilyloxy)-6-methylbenzene (**5a**)

2-[4,5-Bis(*tert*-butyldimethylsilyloxy)-2-methylphenyl]-4,4,5,5-tetramethyl-1,3,2-dioxaborolane (**6a**)

3-Bromo-5-methoxyphenol (**8**)

1-Bromo-3-(*tert*-butyldimethylsilyloxy)-5-methoxybenzene (**9a**)

3',4,5-Tris(*tert*-butyldimethylsilyloxy)-5'-methoxy-2-methyl-1,1'-biphenyl (**10a**)

4-Bromo-5-methylbenzene-1,2-diol (**4**)

4,5-Dibenzyloxy-1-bromo-2-methylbenzene (**5b**)

2-(4,5-Dibenzyloxy-2-methylphenyl)-4,4,5,5-tetramethyl-1,3,2-dioxaborolane (**6b**)

1-(Benzyloxy)-3-bromo-5-methoxybenzene (**9b**)

3',4,5-Tris(benzyloxy)-5'-methoxy-2-methyl-1,1'-biphenyl (**10b**)

5'-Methoxy-6-methyl-[1,1'-biphenyl]-3,3',4-triol; Decarboxyaltenusin (**1**)

Cell Culture and Cytotoxicity Studies

## B. NMR spectra

1. **1-Bromo-3,4-bis(*tert*-butyldimethylsilyloxy)-6-methylbenzene (5a)**
   $^{1}$H NMR (400 MHz, $CDCl_3$)
   $^{13}$C NMR (100 MHz, $CDCl_3$)
2. **2-[4,5-Bis(*tert*-butyldimethylsilyloxy)-2-methylphenyl]-4,4,5,5-tetramethyl-1,3,2-dioxaborolane (6a)**
   $^{1}$H NMR (400 MHz, $CDCl_3$)
   $^{13}$C NMR (100 MHz, $CDCl_3$)
3. **1-Bromo-3-(*tert*-butyldimethylsilyloxy)-5-methoxybenzene (9a)**
   $^{1}$H NMR (400 MHz, $CDCl_3$)
   $^{13}$C NMR (100 MHz, $CDCl_3$)
4. **3',4,5-Tris(*tert*-butyldimethylsilyloxy)-5'-methoxy-2-methyl-1,1'-biphenyl (10a)**
   $^{1}$H NMR (400 MHz, $CDCl_3$)
   $^{13}$C NMR (400 MHz, $CDCl_3$)
5. **4,5-Dibenzyloxy-1-bromo-2-methylbenzene (5b)**
   $^{1}$H NMR (400 MHz, $CDCl_3$)
   $^{13}$C NMR (100 MHz, $CDCl_3$)

6. **2-(4,5-Dibenzyloxy-2-methylphenyl)-4,4,5,5-tetramethyl-1,3,2-dioxaborolane (6b)**
   $^{1}$H NMR (400 MHz, $CDCl_3$)
   $^{13}$C NMR (100 MHz, $CDCl_3$)

7. **1-(Benzyloxy)-3-bromo-5-methoxybenzene (9b)**
   $^{1}$H NMR (400 MHz, $CDCl_3$)
   $^{13}$C NMR (100 MHz, $CDCl_3$)

8. **3’,4,5-Tris(benzyloxy)-5’-methoxy-2-methyl-1,1’-biphenyl (10b)**
   $^{1}$H NMR (400 MHz, $CDCl_3$)
   $^{13}$C NMR (100 MHz, $CDCl_3$)

9. **5’-Methoxy-6-methyl-[1,1’-biphenyl]-3,3’,4-triol; decarboxyaltenusin (1)**
   $^{1}$H NMR (400 MHz, DMSO-$d_6$)
   $^{13}$C NMR (100 MHz, DMSO-$d_6$)
   HSQC (100 MHz, DMSO-$d_6$)
   COSY (100 MHz, DMSO-$d_6$)
   HMBC (100 MHz, DMSO-$d_6$)

## A. Experimental procedures

### *General*

THF and dioxane were distilled from sodium with benzophenone ketyl radical as indicator. All moisture-sensitive reactions were carried out under $O_2$-free argon using oven-dried glassware and a vacuum line. Flash column chromatography [1] was carried out using Merck $SiO_2$ 60 (230–400 mesh) and thin layer chromatography (TLC) was carried out using commercially available Merck $F_{254}$ pre-coated sheets. $^1H$ and $^{13}C$ NMR spectra were recorded with Bruker Avance 300, 400, or 600 instruments. Chemical shifts are given in ppm and are referenced by using the residual signals of the solvent as internal standard. IR spectra were recorded with a Bruker Alpha spectrometer and mass spectra were recorded with a Finnigan MAT-95 mass spectrometer.

### *1,2-Bis(tert-butyldimethylsilyloxy)-4-methylbenzene (3)*

According to a published procedure [2] DMAP (600 mg, 4.91 mmol), imidazole (13.3 g, 196 mmol), and *t*-$BuMe_2SiCl$ (16.7 g, 111 mmol) were added to a solution of 4-methylbenzene-1,2-diol (**2**, 5.17 g, 41.6 mmol) in DMF (150 mL) and the mixture was stirred for 4 h at 50 °C. Then, a saturated aqueous $NaHCO_3$ solution (200 mL) was added and the mixture was extracted with $Et_2O$ (4 × 200 mL). The combined organic layers were washed with aqueous HCl solution (1 M, 100 mL), dried ($Na_2SO_4$), and concentrated at reduced pressure to yield **3** as a yellowish oil (14.2 g, 40.3 mmol, 96%). $^1H$ NMR (300 MHz, $CDCl_3$): $\delta$ (ppm) = 0.21 (s, 6 H, $SiMe_2$), 0.22, (s, 6 H, $SiMe_2$), 1.01 (s, 9 H, Si*t*Bu), 1.02 (s, 9 H, Si*t*Bu), 2.25 (s, 3 H, Ar*Me*), 6.64 (m, 2 H, 2×Ar*H*), 6.74 (d, $^3J$ = 8.0 Hz, 1 H, Ar*H*). These NMR data are in agreement with published data [2].

---

1. Still, W. C.; Kahn, M.; Mitra, A. *J. Org. Chem.* **1978**, *43*, 2923–2925.
2. Berkowitz, D. B.; Smith, M. K. *J. Org. Chem.* **1995**, *60*, 1233–1238.

***1-Bromo-3,4-bis(tert-butyldimethylsilyloxy)-6-methylbenzene (5a)***

In analogy to a published procedure [3] a solution of NBS (4.14 g, 23.3. mmol) in MeCN (60 mL) was added at 0 °C to a solution of protected catechol **3** (7.71 g, 21.9 mmol) in MeCN (75 mL) and stirring was continued for 72 h at room temperature. Then, $H_2O$ (200 mL) was added and the mixture was extracted with hexanes (3 × 100 mL). The combined organic layers were dried ($Na_2SO_4$) and concentrated at reduced pressure to yield **5a** as a red oil (9.09 g, 21.1 mmol, 96%). $^1$H NMR (400 MHz, $CDCl_3$): $\delta$ (ppm) = 0.19 (s, 6 H, $SiMe_2$), 0.20 (s, 6 H, $SiMe_2$), 0.99 (s, 18 H, 2×Si*t*Bu), 2.26 (s, 3 H, Ar*Me*), 6.70 (s, 1 H, Ar*H*), 6.95 (s, 1 H, Ar*H*); $^{13}$C NMR (100 MHz, $CDCl_3$): $\delta$ (ppm) = –4.03 (SiMe), –3.98 (SiMe), 18.6 (C), 22.3 ($CH_3$), 26.1 ($CH_3$), 115.0 (C), 123.1 (CH), 124.6 (CH), 130.5 (C), 145.7 (C), 146.2 (C), signals partly covered; IR (ATR): $\tilde{\upsilon}$ ($cm^{-1}$) = 2928 (w), 2858 (w), 1495 (m), 1305 (m), 1253 (m), 994 (w), 910 (m), 836 (m), 780 (m); MS (EI, 140 °C): *m/z* (%) = 430 (4) [$M^+$], 375 (10), 373 (9), 115 (38), 73 (100), 69 (13); HRMS (EI): found 430.1355. $C_{19}H_{35}O_2{}^{79}Br^{28}Si_2$ requires 430.1354.

***2-[4,5-Bis(tert-butyldimethylsilyloxy)-2-methylphenyl]-4,4,5,5-tetramethyl-1,3,2-dioxaborolane (6a)***

Bromide **5a** (5.00 g, 11.6 mmol) was dissolved under an argon atmosphere in THF (150 mL) and the solution was cooled to −78 °C. Then, *n*-BuLi (2.5M in hexanes, 7.00 mL, 17.5 mmol) was added via a syringe and the mixture was stirred for 45 min at that temperature. 2-Isopropoxy-4,4,5,5-tetramethyl-1,3,2-dioxaborolane (4.8 mL, 4.38 g, 23.5 mmol) was added and the temperature was raised to room temperature overnight. $H_2O$ (500 mL) and brine (150 mL) were added and the organic layer was dried ($Na_2SO_4$), concentrated at reduced pressure, and purified by column chromatography (silica gel, cyclohexane/EtOAc 50:1, with 3% $Et_3N$) to yield **6a** as a yellowish, slowly solidifying oil (3.15 g, 6.57 mmol, 57%). $R_f$ = 0.66

3. Kohler, D.; Podlech, J. *Eur. J. Org. Chem.* **2019**, 1748–1753.

(cyclohexane/EtOAc 10:1); $^{1}$H NMR (400 MHz, $CDCl_3$): $\delta$ (ppm) = 0.20 (s, 6 H, $SiMe_2$), 0.21 (s, 6 H, $SiMe_2$), 0.99 (s, 9 H, Si*t*Bu), 1.00 (s, 9 H, Si*t*Bu), 1.32 (s, 12 H, $Me_2CCMe_2$), 2.42 (s, 3 H, Ar*Me*), 6.64 (s, 1 H, Ar*H*), 7.24 (s, 1 H, Ar*H*); $^{13}$C NMR (100 MHz, $CDCl_3$): $\delta$ (ppm) = –4.0 ($SiCH_3$), –3.9 ($SiCH_3$), 18.6 (C), 18.7 (C), 21.6 ($CH_3$), 25.0 ($CH_3$), 26.1 ($CH_3$), 26.2 ($CH_3$), 83.2 (C), 123.0 (CH), 128.7 (CH), 138.8 (C), 144.0 (C), 149.1 (C); IR (ATR): $\tilde{\upsilon}$ ($cm^{-1}$) = 2928 (m), 2858 (w), 1504 (m), 1471 (w), 1397 (m), 1334 (m), 1305 (m), 1249 (m), 1142 (m), 835 (s), 778 (s); MS (EI, 90 °C): *m/z* (%) = 479 (12), 478 (38) [$M^+$], 477 (8), 322 (62), 321 (100), 320 (52), 231 (24), 181 (100), 131 (100), 100 (27), 73 (100); HRMS (EI): found 478.3100. $C_{25}H_{47}O_4{}^{11}B^{28}Si_2$ requires 478.3101.

### *3-Bromo-5-methoxyphenol (8)*

In slight variation of a published protocol [4] 1-bromo-3,5-dimethoxybenzene (4.23 g, 19.4 mmol) was dissolved under an argon atmosphere in $CH_2Cl_2$ (30 mL) and cooled to −78 °C. $BBr_3$ (1 M in $CH_2Cl_2$, 17 mL, 17.0 mmol) was added dropwise and the mixture was stirred for 18 h while the temperature raised to room temperature. Then, $H_2O$ (40 mL) was added slowly at −78 °C and the mixture was again warmed to room temperature and stirring was continued until clearing of the phases. The aqueous layer was extracted with $Et_2O$ (2 × 50 mL) and the combined organic layers were washed with saturated $NaHCO_3$ solution, dried ($MgSO_4$), concentrated at reduced pressure, and purified by column chromatography (silica gel, cyclohexane/EtOAc 3:1 → 1:1) to yield **8** (2.78 g, 13.7 mmol, 71%) and 5-bromoresorcinol (900 mg, 4.76 mmol, 25%) as colorless solids. **8**: $R_f$ = 0.26 (cyclohexane/EtOAc 3:1); $^{1}$H NMR (300 MHz, $CDCl_3$): $\delta$ (ppm) = 3.76 (s, 3 H, OMe), 5.01 (s, 1 H, OH), 6.33 (m, 1 H, Ar*H*), 6.61, (m, 1 H, Ar*H*), 6.66 (m, 1 H, Ar*H*). These NMR data are in agreement with published data [4].

---

4. Lindgren, A. E. G.; Öberg, C. T.; Hillgren, J. M.; Elofsson, M. *Eur. J. Org. Chem.* **2016**, 426–429.

5-Bromoresorcinol: $R_f$ = 0.08 (hexanes/EtOAc 3:1); $^1$H NMR (300 MHz, acetone-$d_6$): $\delta$ (ppm) = 6.33 (m, 1 H, 2-H), 6.52 (m, 2 H, 4-H, 6-H), 8.63 (s, 2 H, OH). These NMR data are in agreement with published data [5].

***1-Bromo-3-(tert-butyldimethylsilyloxy)-5-methoxybenzene (9a)***

*t*-$BuMe_2SiCl$ (2.58 g, 17.1 mmol), DMAP (29.0 mg, 0.237 mmol), and imidazole (2.27 g, 33.3 mmol) were added successively to a solution of bromide **8** (2.08 g, 10.2 mmol) in DMF (50 mL). The mixture was stirred for 4 h at 55 °C and cooled to room temperature. Then, $H_2O$ (40 mL) and saturated $NaHCO_3$ solution (50 mL) were added and the mixture was extracted with $Et_2O$ (3 × 50 mL). The combined organic layers were dried ($Na_2SO_4$), concentrated at reduced pressure, and purified by column chromatography (silica gel, cyclohexane with 2.3% $Et_3N$) to yield **9a** as a colorless oil (2.37 g, 7.48 mmol, 73%). $R_f$ = 0.35 (cyclohexane); $^1$H NMR (400 MHz, $CDCl_3$): $\delta$ (ppm) = 0.20 (s, 6 H, $SiMe_2$), 0.97 (s, 9 H, Si*t*Bu), 3.75 (s, 3 H, OMe), 6.32 (t, $^4J$ = 2.2 Hz, 1 H, Ar*H*), 6.61 (t, $^4J$ = 1.9 Hz, 1 H, Ar*H*), 6.68 (t, $^4J$ = 1.9 Hz, 1 H, Ar*H*); $^{13}$C NMR (100 MHz, $CDCl_3$): $\delta$ (ppm) = –4.3 ($SiMe_2$), 18.3 (C), 25.7 ($CH_3$), 55.6 ($CH_3$), 105.7 (CH), 110.5 (CH), 116.3 (CH), 122.7 (C), 157.4 (C), 161.2 (C); IR (ATR): $\tilde{\upsilon}$ ($cm^{-1}$) = 2929 (w), 2856 (w), 1592 (m), 1566 (m), 1441 (m), 1424 (m), 1315 (w), 1288 (w), 1157 (m), 1051 (m), 809 (m), 780 (w); MS (EI, 70 °C): *m/z* (%) = 318 (26), 316 (25) [$M^+$], 261 (98), 259 (100), 180 (67), 137 (38), 75 (42), 73 (99), 59 (49), 57 (41); HRMS (EI): found 316.0489. $C_{13}H_{21}O_2{}^{79}Br^{28}Si$ requires 316.0488. No spectroscopic data for comparison were given in the literature for this known compound [6].

---

5. Wu, X.; Zhou, J.; Snider, B. B. *Angew. Chem.* **2009**, *121*, 1309–1312; *Angew. Chem. Int. Ed.* **2009**, *48*, 1283–1286.
6. Wada, Y.; Matsumoto, A.; Asano, K.; Matsubara, S. *RSC Adv.* **2019**, *9*, 31654–31658.

***3',4,5-Tris(tert-butyldimethylsilyloxy)-5'-methoxy-2-methyl-1,1'-biphenyl (10a)***

Boronate **6a** (2.42 g, 5.05 mmol), bromide **9a** (1.99 g, 6.27 mmol), $Pd(OAc)_2$ (40 mg, 0.18 mmol), SPhos (151 mg, 0.366 mmol), and $Cs_2CO_3$ (5.92 g, 18.1 mmol) were dissolved in a 500 mL Schlenk flask under an argon atmosphere in degassed dioxane/$H_2O$ 7:1 (200 mL). The mixture was heated for 18 h at 70 °C, $H_2O$ (300 mL) was added, and the mixture was extracted with EtOAc (4 × 100 mL). The combined organic layers were washed with brine (150 mL), dried ($Na_2SO_4$), concentrated at reduced pressure, and purified by column chromatography (silica gel, cyclohexane with 2.5% $Et_3N$) to yield a non-separable mixture of **10a** and a non-identified by-product (2.91 g, 4.95 mmol, 98%). $R_f$ = 0.47 (cyclohexane); $^1$H NMR (600 MHz, $CDCl_3$): $\delta$ (ppm) = 0.20 (s, 6 H, $SiMe_2$), 0.22 (s, 6 H, $SiMe_2$), 0.23 (s, 6 H, $SiMe_2$), 0.99 (s, 9 H, Si*t*Bu), 0.99 (s, 9 H, Si*t*Bu), 1.02 (s, 9 H, Si*t*Bu), 2.16 (s, 3 H, Ar*Me*), 3.79 (s, 3 H, OMe), 6.36–6.39 (m, 2 H, Ar*H*), 6.44 (s, 1, Ar*H*), 6.69 (s, 1 H, Ar*H*), 6.71 (s, 1 H, Ar*H*); $^{13}$C NMR (150 MHz, $CDCl_3$): $\delta$ (ppm) = –4.2 (2×$SiMe_2$), –3.9 ($SiMe_2$), 18.4 (C), 18.6 (C), 18.6 (C), 20.0 ($CH_3$), 25.9 ($CH_3$), 26.1 ($CH_3$), 26.1 ($CH_3$), 55.4 ($CH_3$), 104.6 (CH), 108.4 (CH), 114.0 (CH), 122.4 (CH), 122.9 (CH), 128.1 (C), 134.9 (C), 143.8 (C), 144.4 (C), 145.9 (C), 156.4 (C), 160.3 (C); IR (ATR): $\tilde{\upsilon}$ ($cm^{-1}$) = 2928 (m), 2856 (w), 1590 (m), 1509 (m), 1290 (m), 1249 (m), 1192 (m), 1158 (m), 1051 (w), 833 (m), 775 (m); MS (EI, 150 °C): *m/z* (%) = 590 (3), 589 (8), 588 (15) [$M^+$], 401 (16), 115 (13), 73 (100); HRMS (EI) found 588.3481. $C_{32}H_{56}O_4{}^{28}Si_3$ requires 588.3483.

***4-Bromo-5-methylbenzene-1,2-diol (4)***

In analogy to a published procedure [3] a solution of NBS (6.33 g, 35.6 mmol) in MeCN (85 mL) was added within 30 min to an ice-cooled solution of 4-methylbenzene-1,2-diol (**2**, 4.10 g, 33.0 mmol) in MeCN (100 mL). The resulting yellow mixture was stirred for 71 h at room temperature while it turned red. It was poured on $H_2O$ (200 mL) and the mixture was extracted with EtOAc (3 × 300 mL). The combined organic layers were dried ($Na_2SO_4$) and

concentrated at reduced pressure to yield **4** as a brown solid (6.71 g, 33.0 mmol, quant.). $R_f$ = 0.65 (cyclohexane/EtOAc 1:1); $^1H$ NMR (300 MHz, DMSO-$d_6$): $\delta$ (ppm) = 2.14 (s, 3 H, $CH_3$), 6.70 (s, 1 H, 3-H or 6-H), 6.88 (s, 1 H, 6-H or 3-H), 9.05 (s, 1 H, OH), 9.14 (s, 1 H, OH); $^{13}C$ NMR (75 MHz, DMSO-$d_6$): $\delta$ (ppm) = 21.5 ($CH_3$), 111.6 (CH), 117.8 (C), 118.6 (CH), 127.1 (C), 144.5 (C), 144.9 (C). The $^1H$ NMR data are in agreement with published data [3,7].

***4,5-Dibenzyloxy-1-bromo-2-methylbenzene (5b)***

In analogy to a published procedure [8] a mixture of brominated catechol **4** (2.17 g, 10.6 mmol), KI (136 mg, 0.82 mmol), $K_2CO_3$ (5.87 g, 42.5 mmol), and BnBr (2.53 mL, 3.64 g, 21.3 mmol) in DMF/acetone 1:2 (40 mL) under an argon atmosphere was stirred for 30 min at room temperature and for 29 h at 70 °C. Then, $H_2O$ (20 mL) was added after cooling and the mixture was extracted with $CHCl_3$ (3 × 70 mL). The combined organic layers were washed with aqueous NaOH solution (5 M, 300 mL) and $H_2O$ (2 × 50 mL), dried ($MgSO_4$), concentrated at reduced pressure, and purified by column chromatography (silica gel, hexane/EtOAc 6:1) to yield **5b** as a colorless solid (3.50 g, 9.14 mmol, 86%) and benzyloxy-4-bromo-5-methylphenol. **5b**: $R_f$ = 0.62 (cyclohexane/EtOAc 6:1); $^1H$ NMR (400 MHz, $CDCl_3$): $\delta$ (ppm) = 2.29 (s, 3 H, $CH_3$), 5.10 (s, 2 H, $CH_2$), 5.12 (s, 2 H, $CH_2$), 6.83 (s, 1 H, 3-H or 6-H), 7.12 (s, 1 H, 6-H or 3-H), 7.30–7.43 (m, 10 H, 2 Ph); $^{13}C$ NMR (100 MHz, $CDCl_3$): $\delta$ (ppm) = 22.5 ($CH_3$), 71.7 ($CH_2$), 71.8 ($CH_2$), 115.5 (CH), 117.5 (C), 119.2 (CH), 127.5 (CH), 127.5 (CH), 128.0 (CH), 128.1 (CH), 128.7 (CH), 130.8 (C), 137.0 (C), 137.1 (C), 147.8 (C), 148.3 (C); IR (ATR): $\tilde{\upsilon}$ ($cm^{-1}$) = 3062 (vw), 3034 (vw), 2874 (vw), 1567 (vw), 1499 (w), 1453 (w), 1382 (w), 1369 (w), 1320

---

7. Alig, L.; Alsenz, J.; Andjelkovic, M.; Bendels, S.; Bénardeau, A.; Bleicher, K.; Bourson, A.; David-Pierson, P.; Guba, W.; Hildbrand, S.; Kube, D.; Lübbers, T.; Mayweg, A. V.; Narquizian, R.; Neidhart, W.; Nettekoven, M.; Plancher, J.-M.; Rocha, C.; Rogers-Evans, M.; Röver, S.; Schneider, G.; Taylor, S.; Waldmeier, P. *J. Med. Chem.* **2008**, *51*, 2115–2127.
8. da Silva Pinto, A. C.; Silva, L. F. R.; Cavalcanti, B. C.; Melo, M. R. S.; Chaves, F. C. M.; Lotufo, L. V. C.; de Moraes, M. O.; de Andrade-Neto, V. F.; Tadei, W. P.; Pessoa, C. O.; Ribeiro Vieirac, P. P.; Pohlit, A. M. *Eur. J. Med. Chem.* **2009**, *44*, 2731–2735.

(w), 1199 (w), 1157 (m), 1008 (w), 965 (w), 914 (w), 848 (w), 747 (w), 719 (w), 696 (m); MS (FAB), *m/z* (%) = 385 (15), 384 (35) [$C_{21}H_{19}{}^{81}BrO_2{}^+$], 383 (17), 382 (34) [$C_{21}H_{19}{}^{79}BrO_2{}^+$], 136 (29), 91 (100) [$C_7H_7{}^+$]; HRMS (FAB) found 382.0570. $C_{21}H_{19}{}^{79}BrO_2{}^+$ requires 382.0568. 2-Benzyloxy-4-bromo-5-methylphenol: $R_f$ = 0.22 (hexanes/EtOAc 19:1); $^1$H NMR (300 MHz, $CDCl_3$): $\delta$ (ppm) = 2.29 (s, 3 H, $CH_3$), 5.05 (s, 4 H, 2×$CH_2$), 5.54 (br s, 1 H, OH), 6.82 (d, $J$ = 10.8 Hz, 1 H, H-3 or H-6), 7.11 (d, $J$ = 9.5 Hz, 1 H, H-6 or H-3), 7.34–7.45 (m, 5 H, Ph).

***2-(4,5-Dibenzyloxy-2-methylphenyl)-4,4,5,5-tetramethyl-1,3,2-dioxaborolane (6b)***

A solution of bis(benzylether) **5b** (100 mg, 0.261 mmol), KOAc (77 mg, 0.78 mmol), Pd(dppf)$Cl_2$·$CH_2Cl_2$ (21 mg, 0.026 mmol), and bis(pinacolato)diboron (133 mg, 0.524 mmol) in anhydrous and degassed dioxane (1.4 mL) under an argon atmosphere was heated to 80 °C for 17 h. The mixture was filtered through a Celite pad and rinsed with EtOAc, concentrated at reduced pressure, and purified by column chromatography (silica gel, cyclohexane/toluene 1:1 → toluene → cyclohexane/EtOAc 4:1) to yield **6b** as a colorless liquid (62 mg, 0.144 mmol, 55%). $R_f$ = 0.17 (cyclohexane/toluene 1:1); $^1$H NMR (300 MHz, $CDCl_3$): $\delta$ (ppm) = 1.35 (s, 12 H, 4×Me), 2.49 (s, 3 H, 2-Me), 5.16 (s, 2 H, $CH_2$) 5.18 (s, 2 H, $CH_2$), 6.79 (s, 1 H, 3-H or 6-H), 7.41 (s, 1 H, 6-H or 3-H), 7.27–7.54 (m, 10 H, 2 Ph); $^{13}$C NMR (100 MHz, $CDCl_3$): $\delta$ (ppm) = 21.9 ($CH_3$), 25.0 (4×$CH_3$), 70.8 ($CH_2$), 71.9 ($CH_2$), 83.4 (2×C), 116.5 (CH), 122.9 (CH), 127.3 (2×CH) 127.8 (2×CH), 127.8 (CH), 127.8 (CH), 128.5 (2×CH), 128.6 (2× CH), 137.4 (C), 137.8 (C), 140.0 (C), 146.2 (C), 151.3 (C), signal of C-1 could not be detected (due to boron’s quadrupole moment); IR (ATR): $\tilde{\upsilon}$ (cm$^{-1}$) = 2972 (w), 1592 (m), 1512 (w), 1456 (w), 1407 (m), 1338 (m), 1300 (m), 1248 (m), 1139 (m), 1011 (m), 867 (m), 847 (m), 738 (m), 724 (m), 691 (m); MS (FAB): *m/z* (%) = 431 (17), 430 (48), 429 (14), 91 (100); HRMS (FAB) found 430.2316. $C_{27}H_{31}{}^{11}BO_4{}^+$ requires 430.2315.

***1-(Benzyloxy)-3-bromo-5-methoxybenzene (9b)***

BnBr (1.17 mL, 9.86 mmol) was added under an argon atmosphere to a solution of bromide **8** (1.61 g, 7.89 mmol) and $K_2CO_3$ (2.70 g, 19.7 mmol) in DMF/acetone 1:2 (60 mL). The mixture was stirred for 43 h at 80 °C and cooled to room temperature. To the brown suspension were added $H_2O$ (100 mL) and 1 M HCl until pH 8. The aqueous layer was extracted with EtOAc (3 × 100 mL) and the combined organic layers were washed with brine (50 mL) and $H_2O$ (100 mL), dried ($MgSO_4$), concentrated at reduced pressure, and purified by column chromatography (silica gel, hexanes/EtOAc 2:1) to yield **9b** as a colorless oil (2.27 g, 7.74 mmol, 98%). $R_f$ = 0.32 (cyclohexane/EtOAc 50:1); $^1$H NMR (300 MHz, $CDCl_3$): $\delta$ (ppm) = 3.77 (s, 3 H, Me), 5.02 (s, 2 H, $CH_2$), 6.47 (t, $J$ = 2.1 Hz, 1 H, Ar-H), 6.69 (t, $J$ = 1.8 Hz, 1 H, Ar-H), 6.77 (t, $J$ = 1.9 Hz, 1 H, Ar-H), 7.33–7.44 (m, 5 H, Ph); $^{13}$C NMR (75 MHz, $CDCl_3$): $\delta$ (ppm) = 55.6 ($CH_3$), 70.4 ($CH_2$), 100.7 (CH), 110.3 (CH), 110.8 (CH), 123.1 (C), 127.7 (CH), 128.3 (CH), 128.8 (CH), 136.5 (C), 160.5 (C), 161.4 (C); IR (ATR): $\tilde{\upsilon}$ (cm$^{-1}$) = 2926 (w), 1595 (m), 1573 (m), 1425 (m), 1378 (m), 1329 (w), 1277 (m), 1192 (m), 1152 (s), 1055 (m), 1024 (m), 812 (m), 735 (m), 696 (m), 675 (m); MS (EI, 20 °C): *m/z* (%) = 294 (13) [$C_{14}H_{13}{}^{81}BrO_2{}^+$], 292 (14) [$C_{14}H_{13}{}^{79}BrO_2{}^+$], 218 (40) [$C_8H_8{}^{81}BrO_2{}^+$], 216 (41) [$C_8H_8{}^{79}BrO_2{}^+$], 108 (26), 107 (10), 105 (10), 92 (11), 91 (100) [$C_7H_7{}^+$], 79 (13), 77 (18), 69 (21), 65 (10), 58 (19), 57 (14); HRMS (EI) found 292.0098. $C_{14}H_{13}O_2{}^{79}Br^+$ requires 292.0099.

***3',4,5-Tris(benzyloxy)-5'-methoxy-2-methyl-1,1'-biphenyl (10b)***

A solution of boronate **6b** (104 mg, 0.242 mmol), bromide **9b** (85 mg, 0.290 mmol), $Cs_2CO_3$ (315 mg, 0.967 mmol), $Pd(OAc)_2$ (2.7 mg, 12 µmol), and 2-dicyclohexylphosphino-2',6'-dimethoxybiphenyl (SPhos, 8.7 mg, 21 µmol) in degassed dioxane/$H_2O$ 7:1 (9.3 mL) was heated for 18 h at 70 °C under an argon atmosphere. After cooling to room temperature $H_2O$ (15 mL) was added and the aqueous phase was extracted with EtOAc (3 × 40 mL). The combined organic layers were washed with brine (75 mL), dried ($Na_2SO_4$), concentrated at

reduced pressure, and purified by column chromatography (silica gel, cyclohexane/EtOAc 50:1 → 15:1) to yield **10b** as a colorless, highly viscous product (111 mg, 0.215 mmol, 89%). $R_f$ = 0.32 (cyclohexane/EtOAc 10:1); $^1$H NMR (300 MHz, $CDCl_3$): $\delta$ (ppm) = 2.16 (s, 3 H, 2-Me), 3.79 (s, 3 H, OMe), 5.05 (s, 2 H, $CH_2$), 5.13 (s, 2 H, $CH_2$), 5.18 (s, 2 H, $CH_2$), 6.42 (dd, $J$ = 2.2, 1.3 Hz, 1 H, Ar-H), 6.47 (dd, $J$ = 2.2, 1.4 Hz, 1 H, Ar-H), 6.52 (t, $J$ = 2.3 Hz, 1 H, Ar-H), 6.84 (s, 1 H, Ar-H), 6.86 (s, 1 H, Ar-H), 7.29–7.50 (m, 15 H, 3 Ph); $^{13}$C NMR (100 MHz, $CDCl_3$) $\delta$ (ppm) = 20.0 ($CH_3$), 55.5 ($CH_3$), 70.2 ($CH_2$), 71.6 ($CH_2$), 71.8 ($CH_2$), 99.9 (CH), 108.1 (CH), 108.4 (CH), 117.2 (CH), 117.3 (CH), 127.5 (2×CH), 127.6 (2×CH), 127.6 (2×CH), 127.8 (CH), 127.9 (CH), 128.1 (CH), 128.5 (C), 128.5 (2×CH), 128.6 (2×CH), 128.7 (2×CH), 134.9 (C), 137.1 (C), 137.6 (2×C), 143.8 (C), 146.8 (C), 148.4 (C), 159.7 (C), 160.5 (C); IR (ATR): $\tilde{\nu}$ ($cm^{-1}$) = 3031 (w), 2924 (w), 1590 (m), 1509 (m), 1453 (m), 1427 (w), 1378 (w), 1252 (m), 1190 (m), 1152 (m), 1065 (m), 1025 (m), 839 (m), 735 (m), 696 (m); MS (FAB): $m/z$ (%) = 517 (11) [$M^+$+1], 516 (15) [$M^+$], 91 (100) [$C_7H_7^+$]; HRMS (FAB) found 516.2293. $C_{35}H_{32}O_4^+$ requires 516.2295.

***5’-Methoxy-6-methyl-[1,1’-biphenyl]-3,3’,4-triol; decarboxyaltenusin (1)***

A mixture of benzyl-protected decarboxyaltenusin **10b** (100 mg, 0.194 mmol) and Pd/C (10%, 22 mg, 0.207 mmol) in THF (10 mL) in an autoclave with $H_2$ pressure (8 bar) was heated at 40 °C for 24 h, cooled, filtered over Celite, dried ($Na_2SO_4$), concentrated at reduced pressure, and purified by column chromatography (silica gel, hexanes/EtOAc 1:1) to yield **1** as a yellow oil (42 mg, 0.171 mmol, 88%). $R_f$ = 0.36 (hexanes/EtOAc 1:1); $^1$H NMR (400 MHz, DMSO-$d_6$): $\delta$ (ppm) = 2.05 (s, 3 H, 6-Me), 3.70 (s, 3 H, OMe), 6.20 (dd, $J$ = 2.1, 1.5 Hz, 1 H, 6’-H), 6.22 (t, $J$ = 1.7 Hz, 1 H, 2’-H), 6.25 (t, $J$ = 2.2 Hz, 1 H, 4’-H), 6.55 (s, 1 H, 2-H), 6.60 (s, 1 H, 5-H), 8.72 (br s, 1 H, 3-OH), 8.78 (br s, 1 H, 4-OH), 9.37 (br s, 1 H, 3’-OH); $^{13}$C NMR (100 MHz, DMSO-$d_6$): $\delta$ (ppm) = 19.4 ($CH_3$, OMe), 54.9 ($CH_3$, 6-Me), 99.2 (CH, C-4’), 105.8 (CH, C-6’), 108.8 (CH, C-2’), 116.7 (CH, C-2), 117.5 (CH, C-5), 124.8 (C, C-6), 132.3 (C, C-1),

142.9 (C, C-1’), 143.6 (C, C-4), 144.3 (C, C-3), 158.0 (C, C-3’), 160.0 (C, C-5’); IR (ATR): $\tilde{\upsilon}$ ($cm^{-1}$) = 3332 (br s), 2919 (m), 2849 (s), 1591 (m), 1519 (s), 1496 (s), 1427 (m); MS (EI, 140 °C): *m/z* (%) = 247 (17), 246 (100) [$M^+$], 245 (19), 197 (10), 73 (11), 69 (18) 57 (14), 55 (15); HRMS (FAB) found 246.0893. $C_{14}H_{14}O_4^+$ requires 246.0892.

***Cell culture and cytotoxicity studies***

HeLa cells (human cervix carcinoma cell line) cells were cultured in DMEM medium (*Gibco*) supplemented with 10% fetal bovine serum (FBS, Gibco) and 1% penicillin/streptavidin (*Gibco*) at 37 °C, 5% $CO_2$. The cells were trypsinized (0.05% trypsin-EDTA, *Gibco*) and seeded into 96 well plates (toxicity assay) at 70% confluency for 24 h.

The medium was removed and the cells were treated with the indicated concentrations of decarboxyaltenusin by diluting the DMSO stock solution in 100 µl DMEM supplemented with 10% FBS and penicillin/streptavidin as described above. The cells were incubated for 72 h at 37 °C, 5% $CO_2$. For the negative control, the cell culture medium was replaced by medium. Eventually, the MTT assay was performed as describe in the manufacturer’s manual (*Promega*). Prior to MTT assay, the positive control was treated with Triton X-100 (1%). After 3 h of incubation with the 15 µL of the MTT reagent the cells were lysed using the Stop Solution to release the blue-purple formazan. The cell viability was determined by measuring the absorbance of the resulting formazan at 595 nm using a multiwell plate reader (SpectraMax ID3, *Molecular Devices*, USA). The values were normalized against the positive and negative control. Experiments were performed with $n = 5$ and standard deviations were calculated using Student’s *t*-test.

## B. NMR Spectra

## 1. 1-Bromo-3,4-bis(*tert*-butyldimethylsilyloxy)-6-methylbenzene (5a)

$^{1}$H NMR (400 MHz, $CDCl_3$)

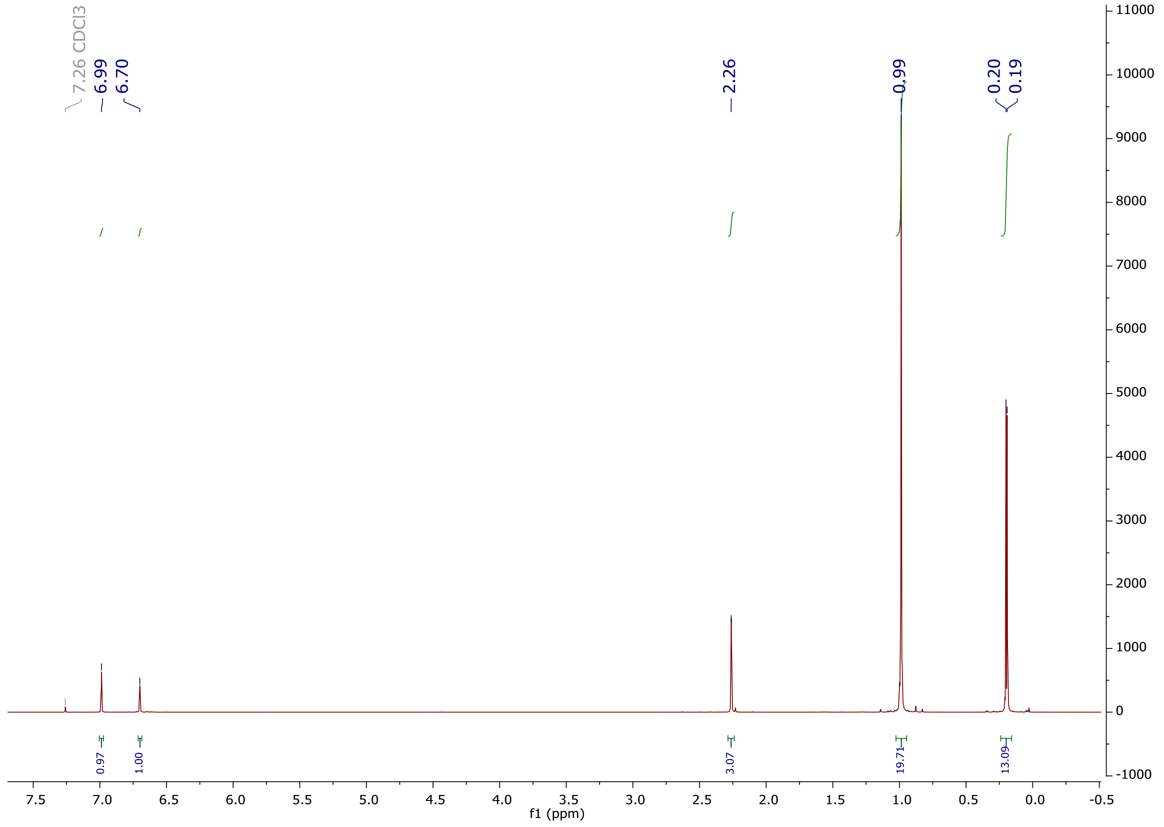


$^{13}$C NMR (100 MHz, $CDCl_3$)

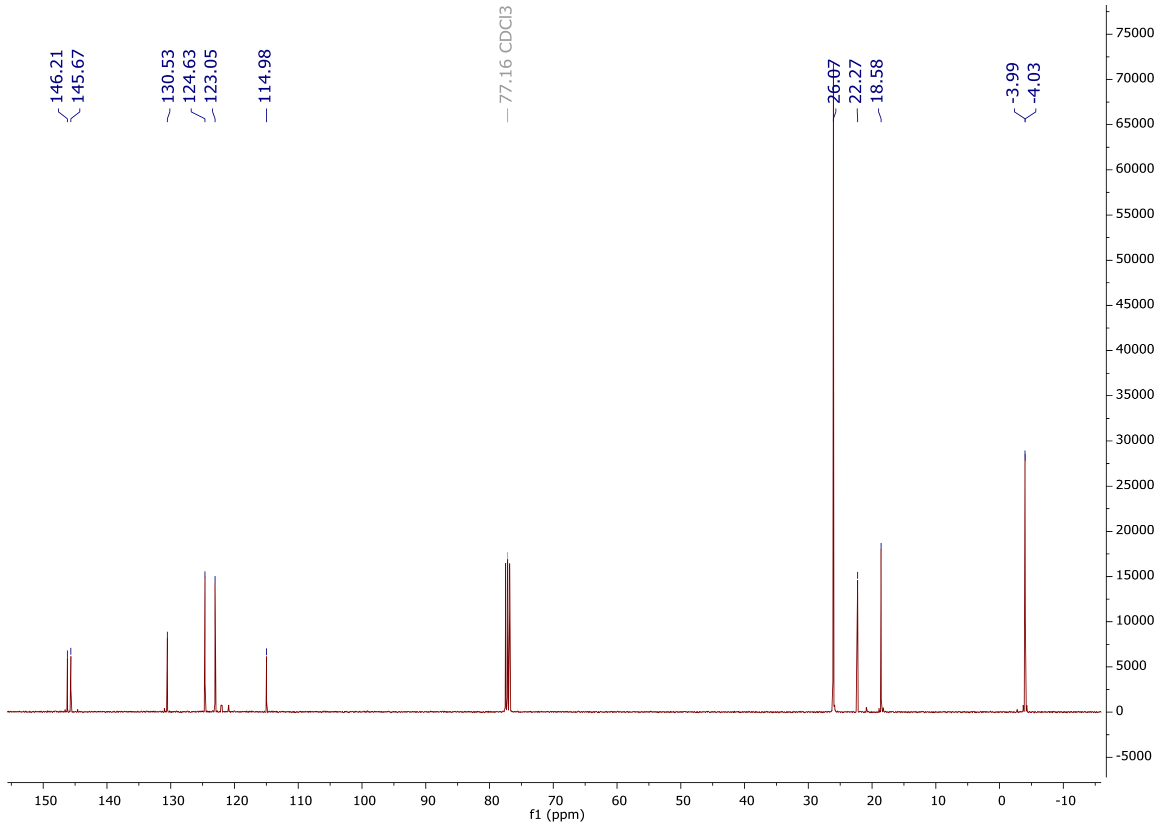

## 2. 2-[4,5-Bis(*tert*-butyldimethylsilyloxy)-2-methylphenyl]-4,4,5,5-tetramethyl-1,3,2-dioxaborolane (6a)

$^{1}$H NMR (400 MHz, $CDCl_3$)

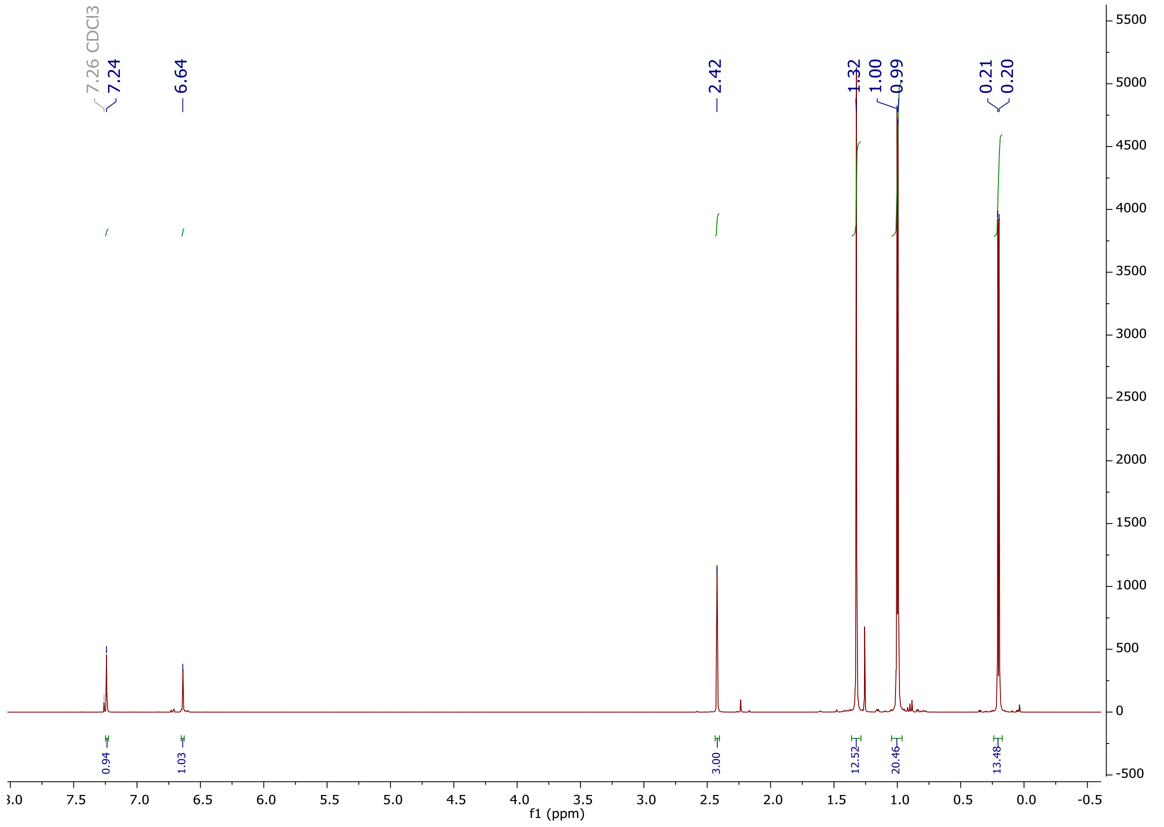


$^{13}$C NMR (100 MHz, $CDCl_3$)

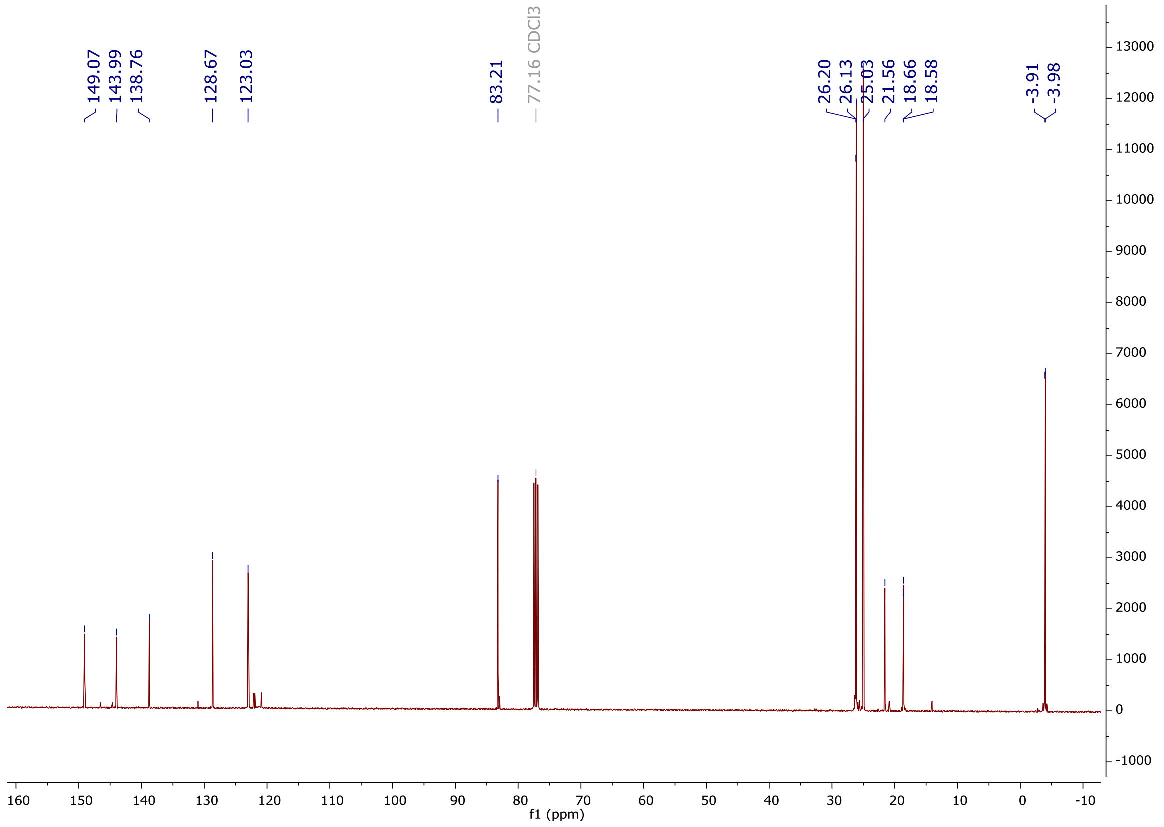

## 3. 1-Bromo-3-(*tert*-butyldimethylsilyloxy)-5-methoxybenzene (9a)

$^{1}$H NMR (400 MHz, $CDCl_3$)

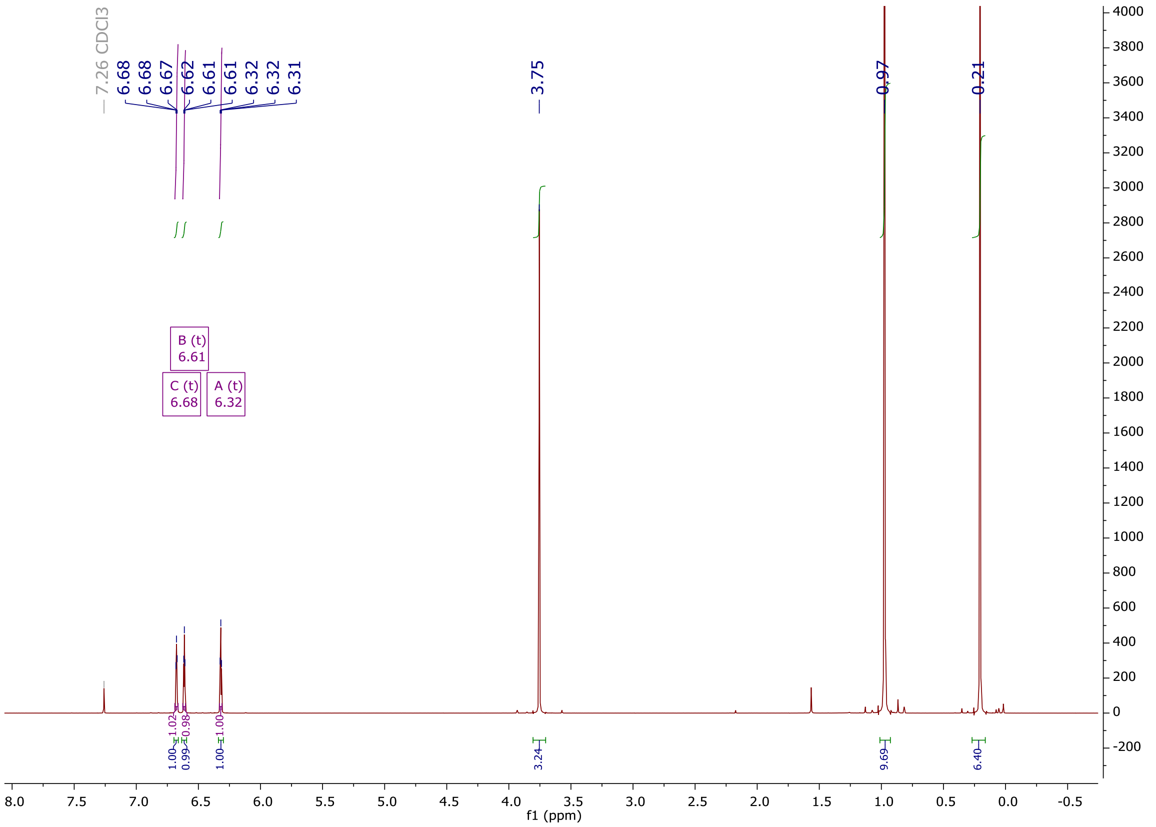


$^{13}$C NMR (100 MHz, $CDCl_3$)

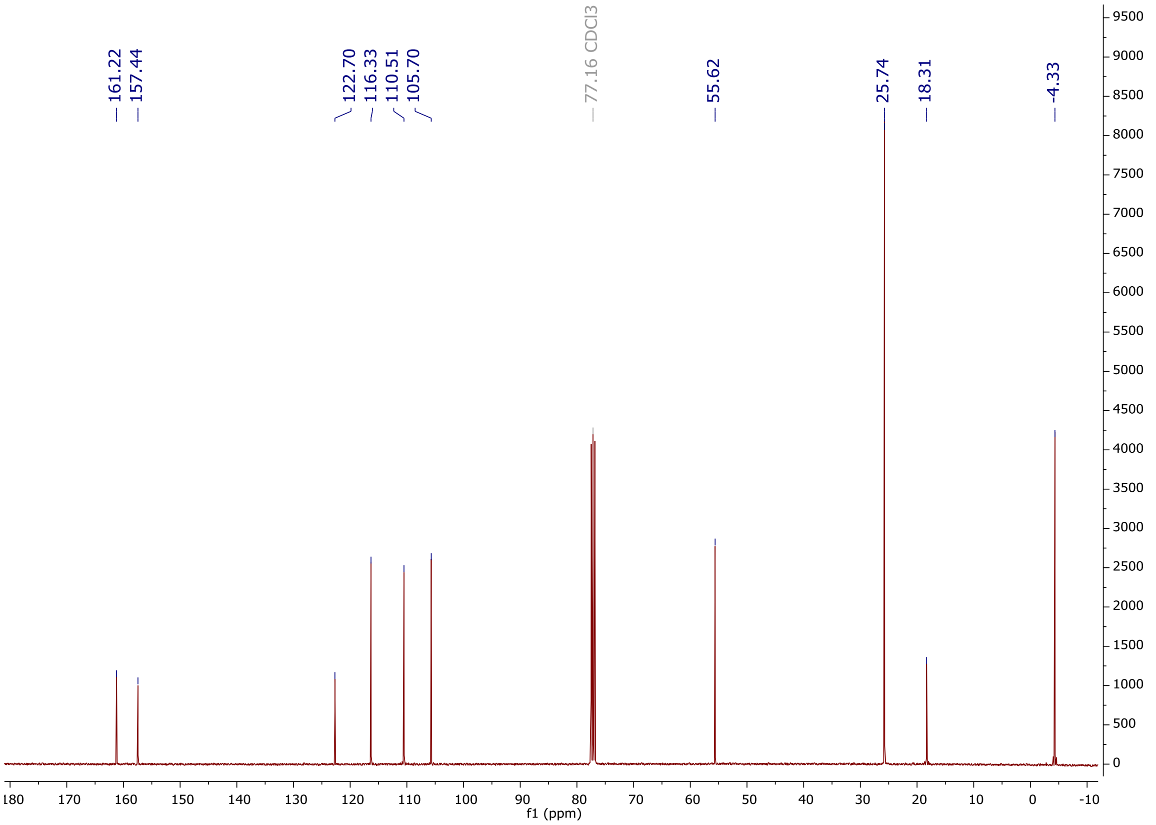

**4. 3',4,5-Tris(*tert*-butyldimethylsilyloxy)-5'-methoxy-2-methyl-1,1'-biphenyl (10a)**

$^{1}$H NMR (400 MHz, $CDCl_3$)

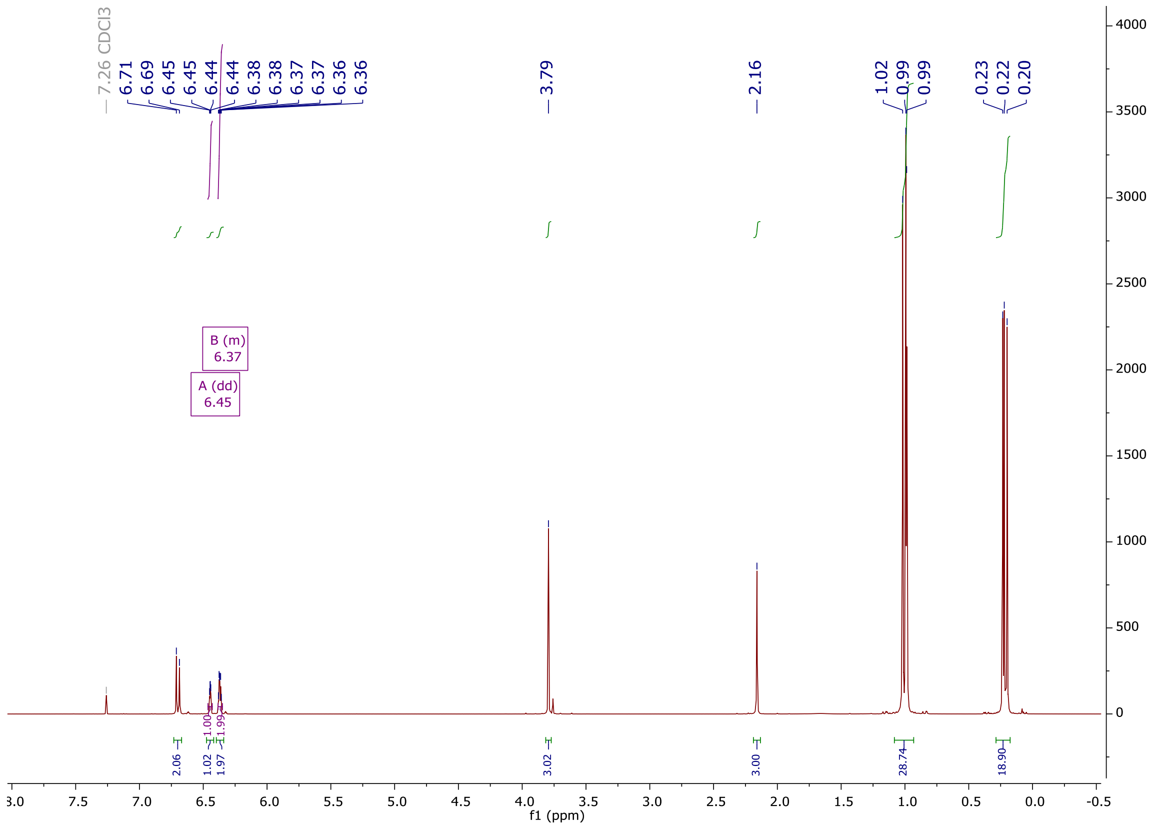

$^{13}$C NMR (400 MHz, $CDCl_3$)

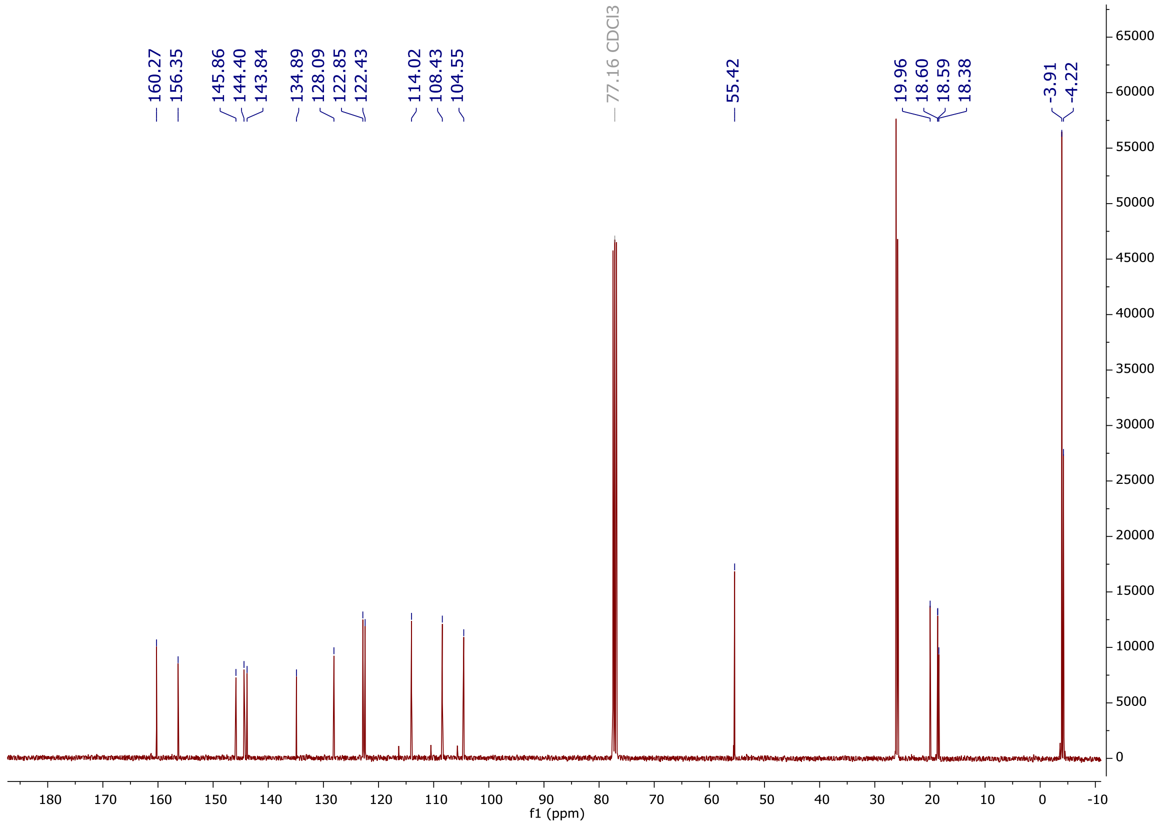

## 5. 4,5-Dibenzyloxy-1-bromo-2-methylbenzene (5b)

$^1$H NMR (400 MHz, $CDCl_3$)

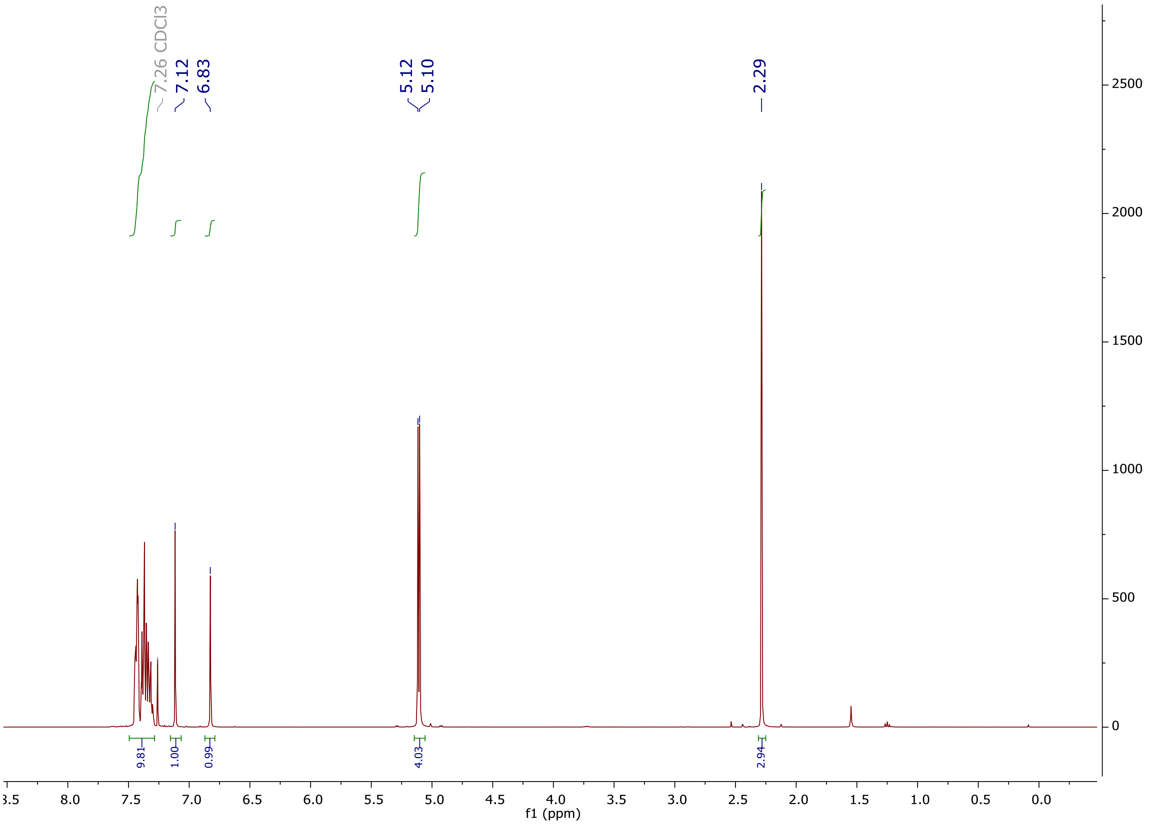


$^{13}$C NMR (100 MHz, $CDCl_3$)

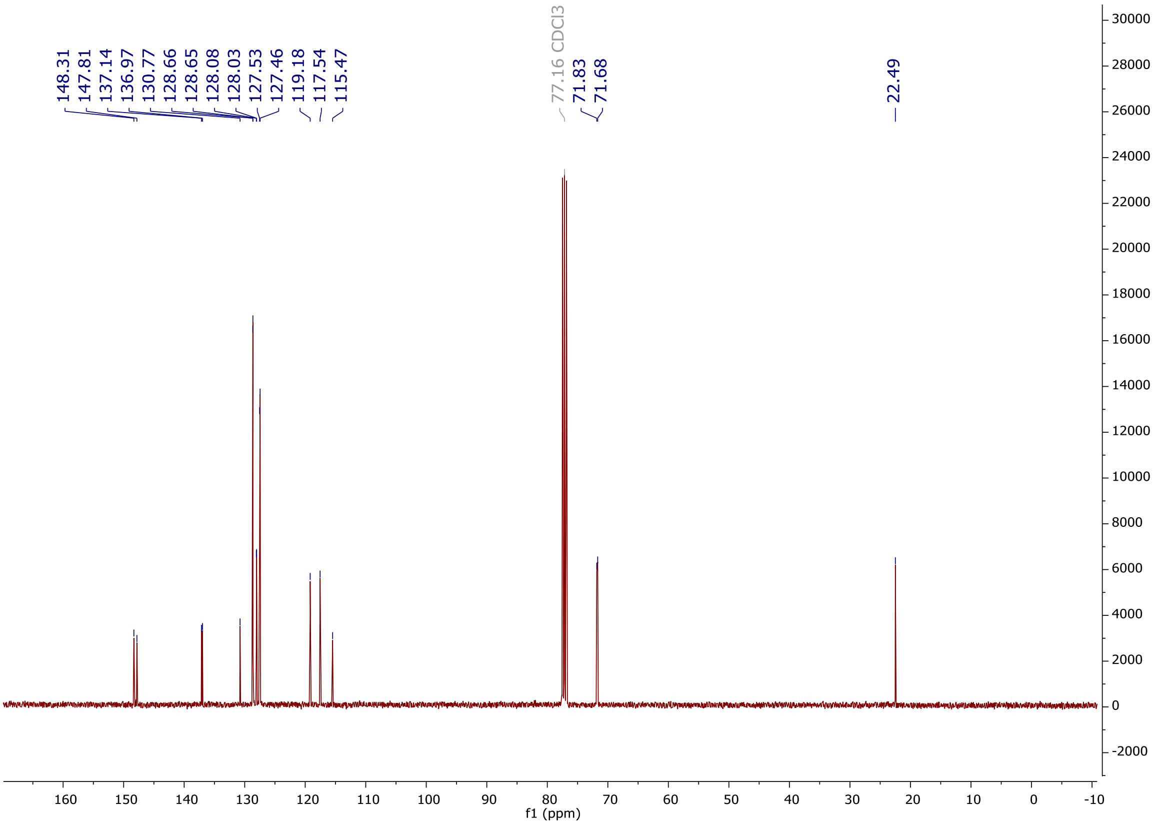

## 6. 2-(4,5-Dibenzyloxy-2-methylphenyl)-4,4,5,5-tetramethyl-1,3,2-dioxaborolane (6b)

$^{1}$H NMR (400 MHz, $CDCl_3$)

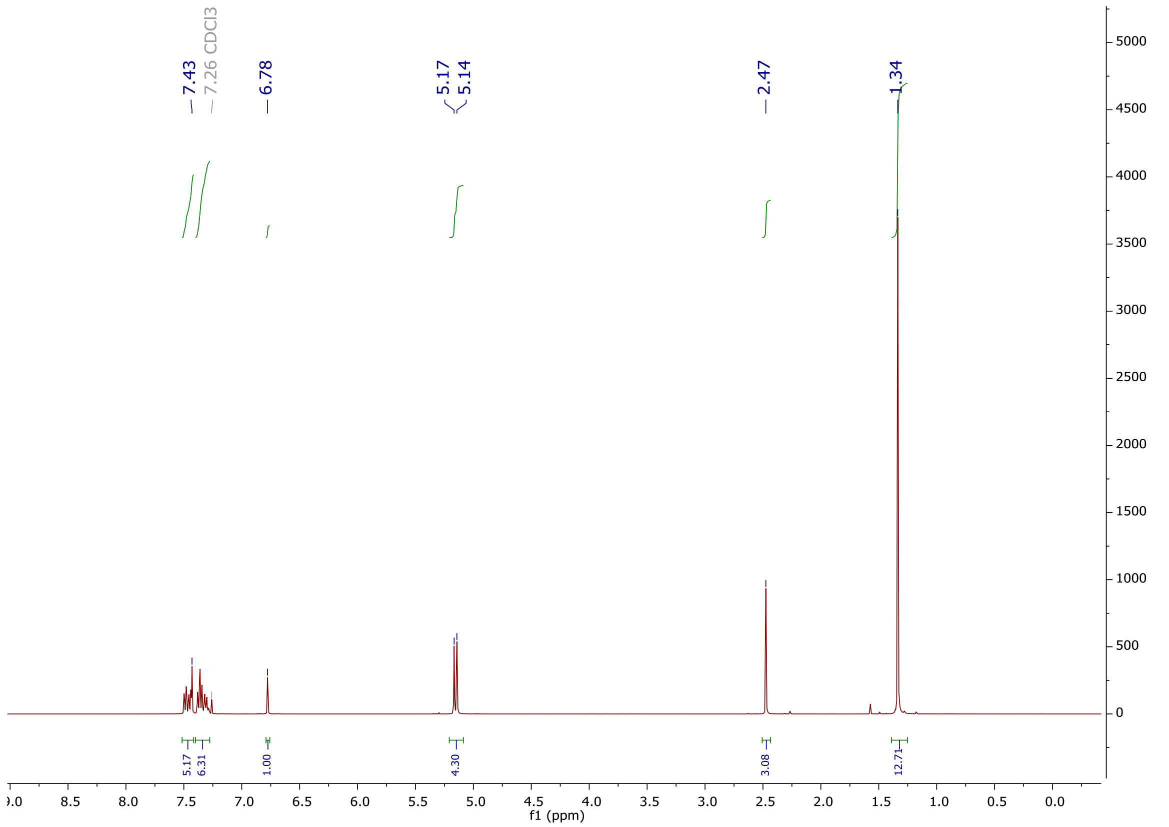


$^{13}$C NMR (100 MHz, $CDCl_3$)

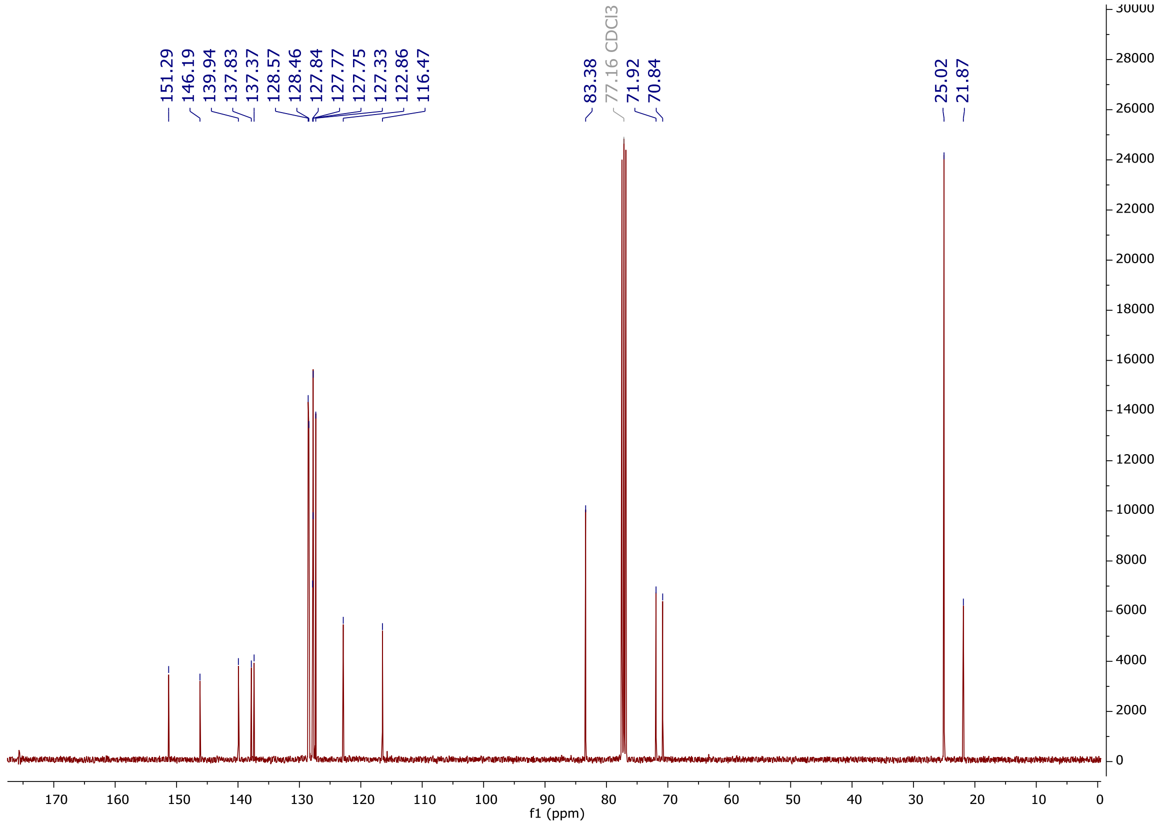

## 7. 1-(Benzyloxy)-3-bromo-5-methoxybenzene (9b)

$^{1}$H NMR (400 MHz, $CDCl_3$)

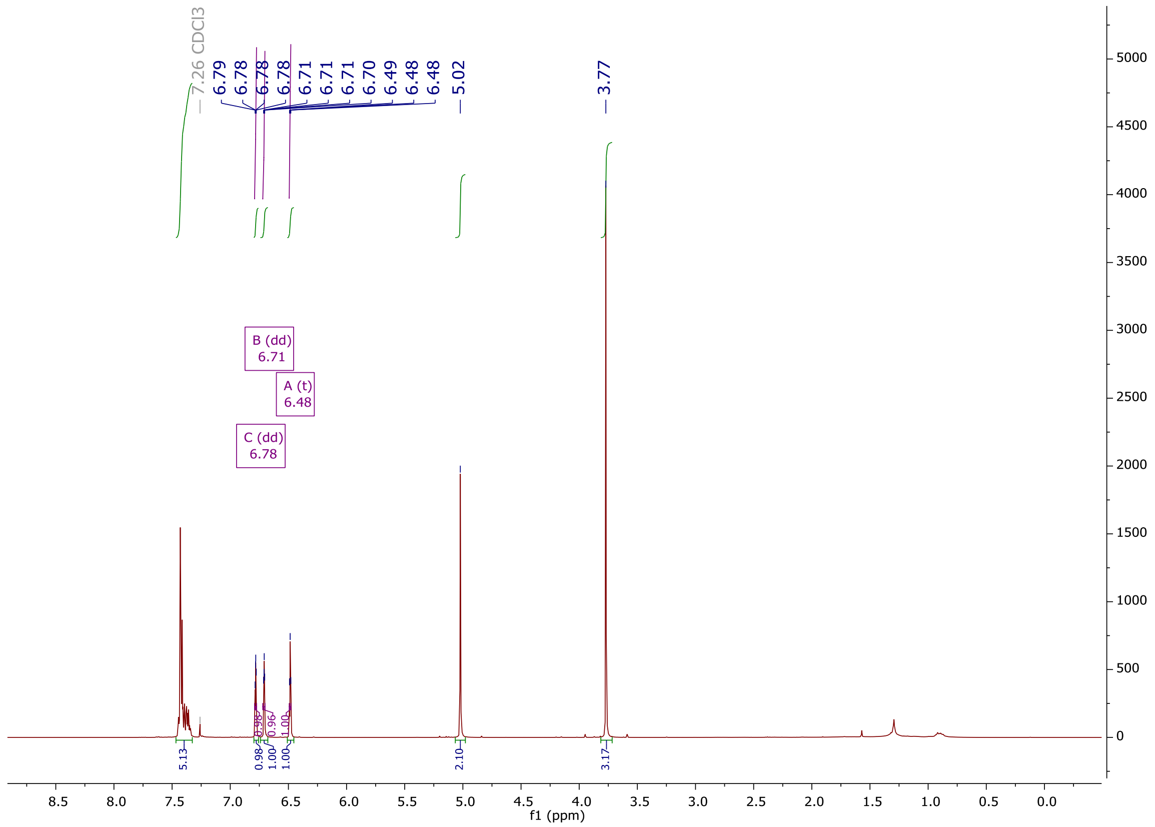


$^{13}$C NMR (100 MHz, $CDCl_3$)

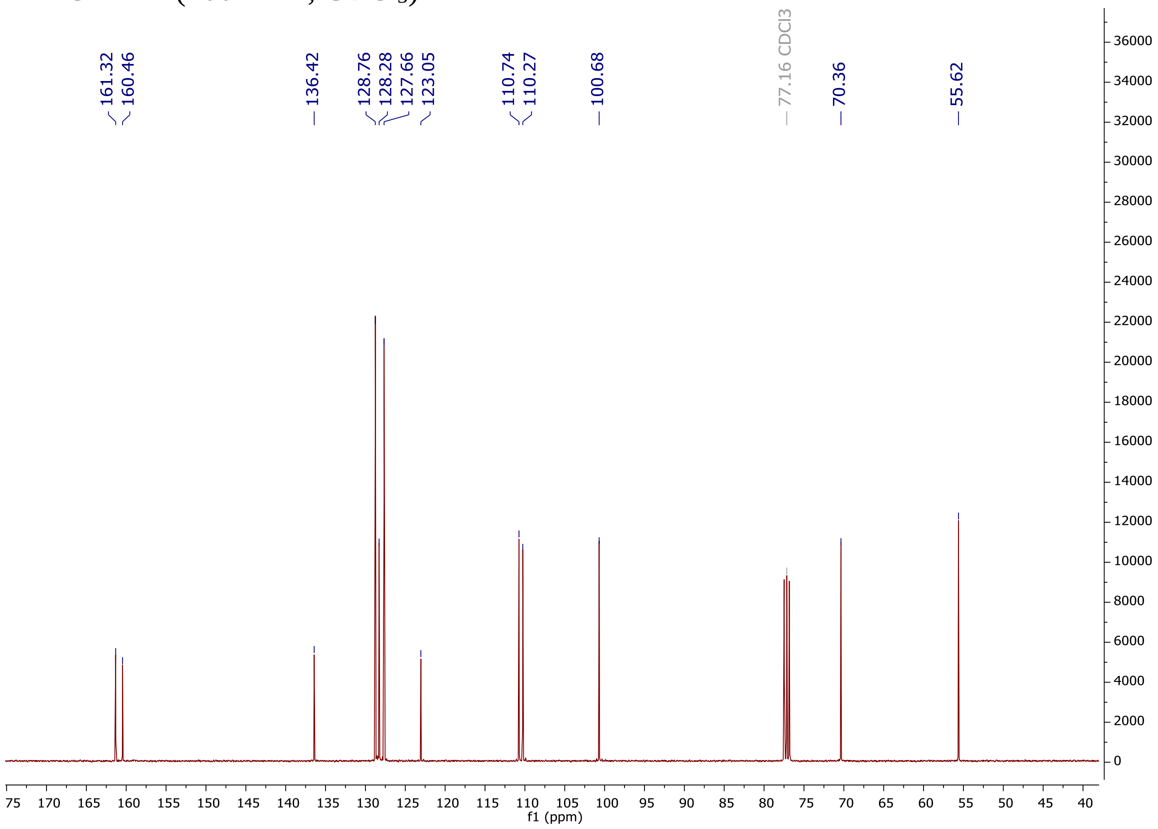

## 8. 3’,4,5-Tris(benzyloxy)-5’-methoxy-2-methyl-1,1’-biphenyl (10b)

$^{1}$H NMR (400 MHz, $CDCl_3$)

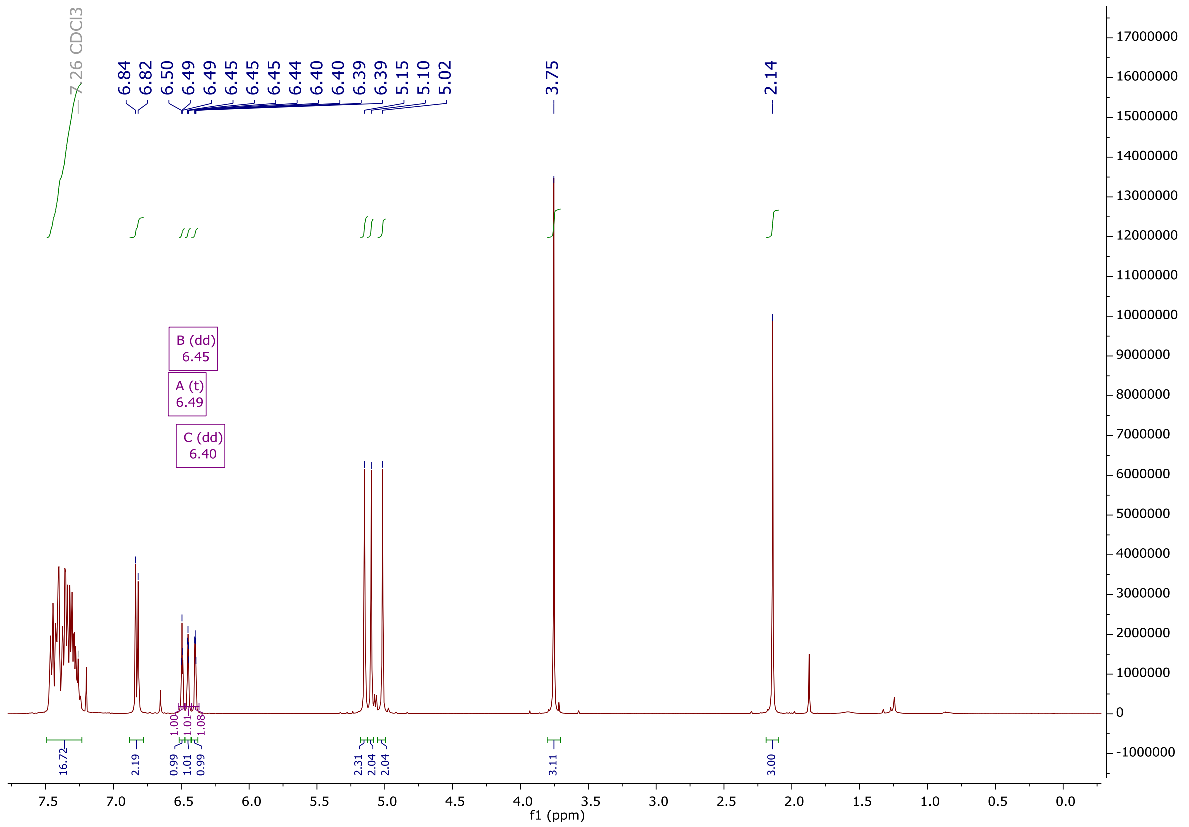


$^{13}$C NMR (100 MHz, $CDCl_3$)

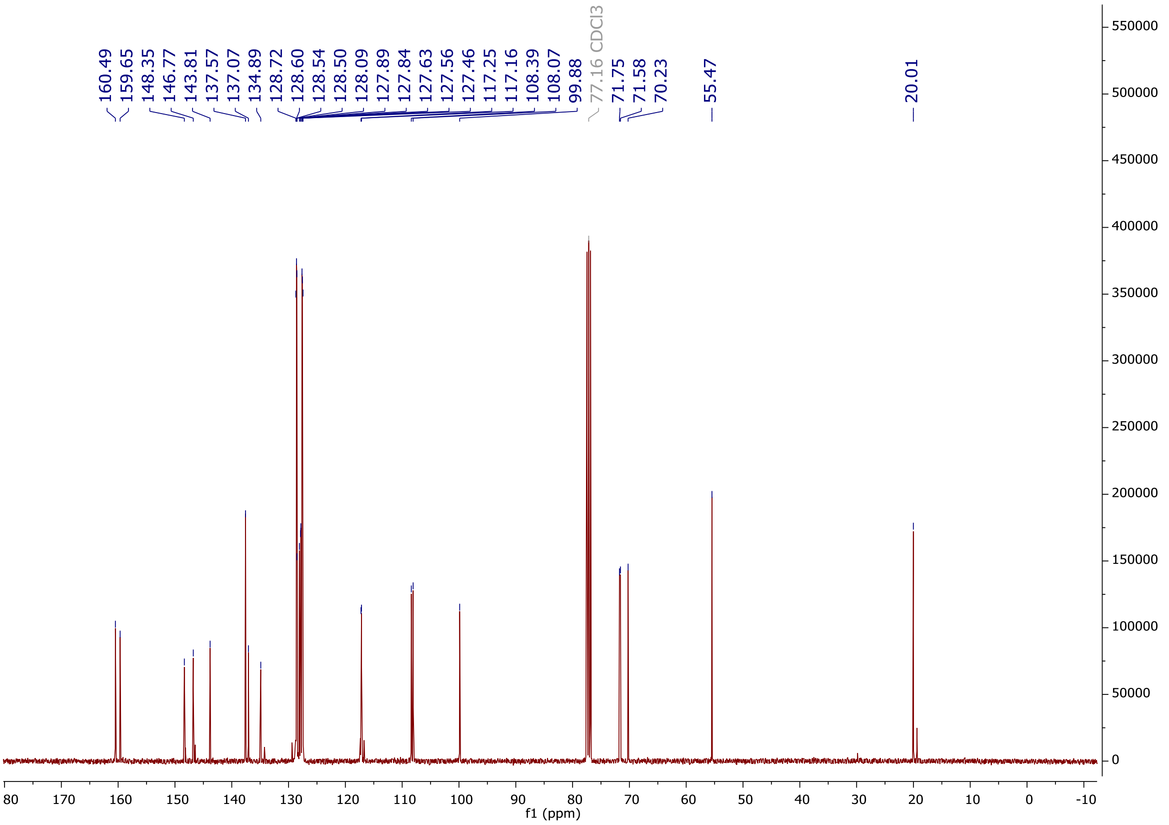

**9. 5’-Methoxy-6-methyl-[1,1’-biphenyl]-3,3’,4-triol; decarboxyaltenusin (1)**

$^{1}$H NMR Spectrum (400 MHz, DMSO-$d_6$)

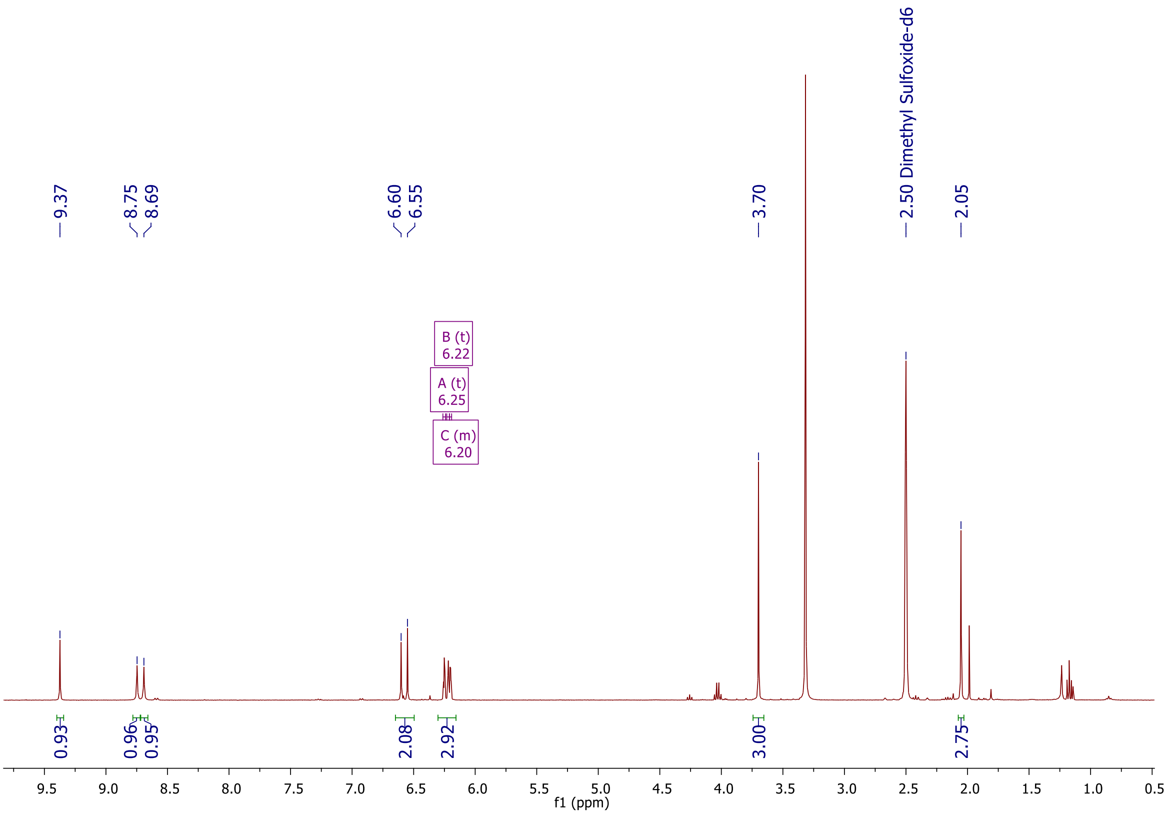


$^{13}$C NMR (100 MHz, DMSO-$d_6$)

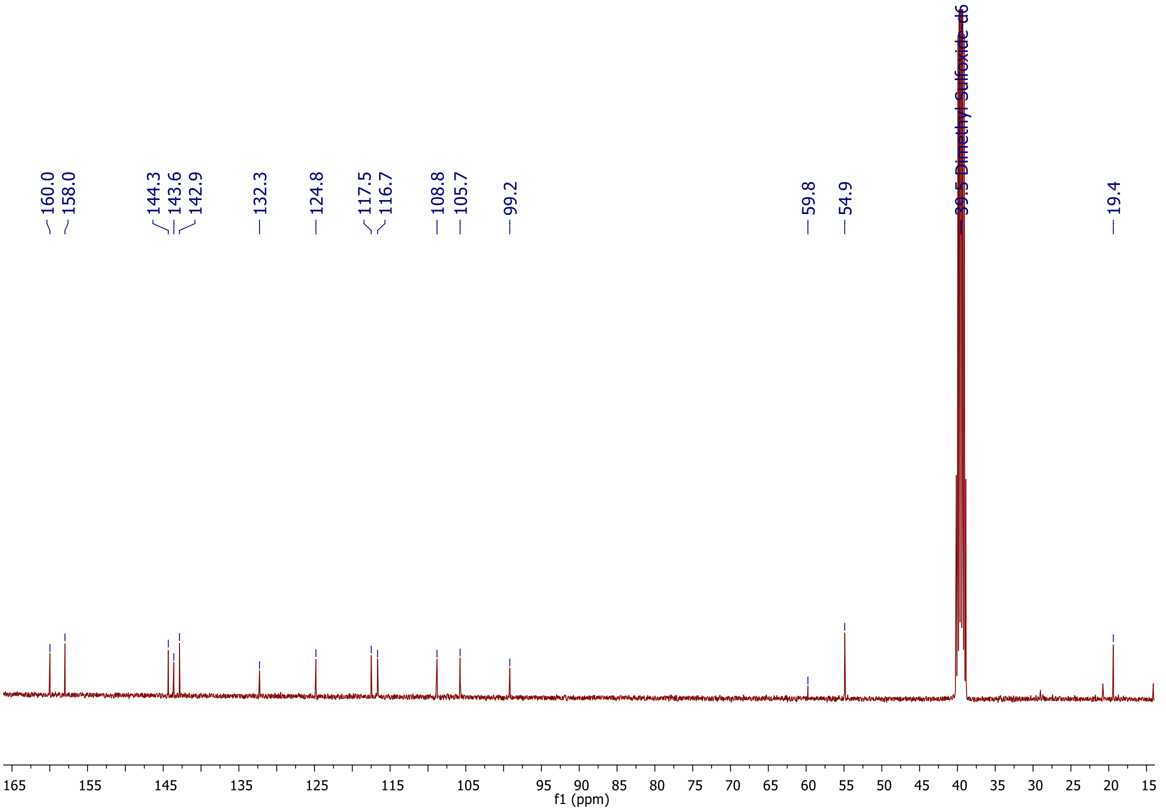

HSQC (100 MHz, DMSO-$d_6$)

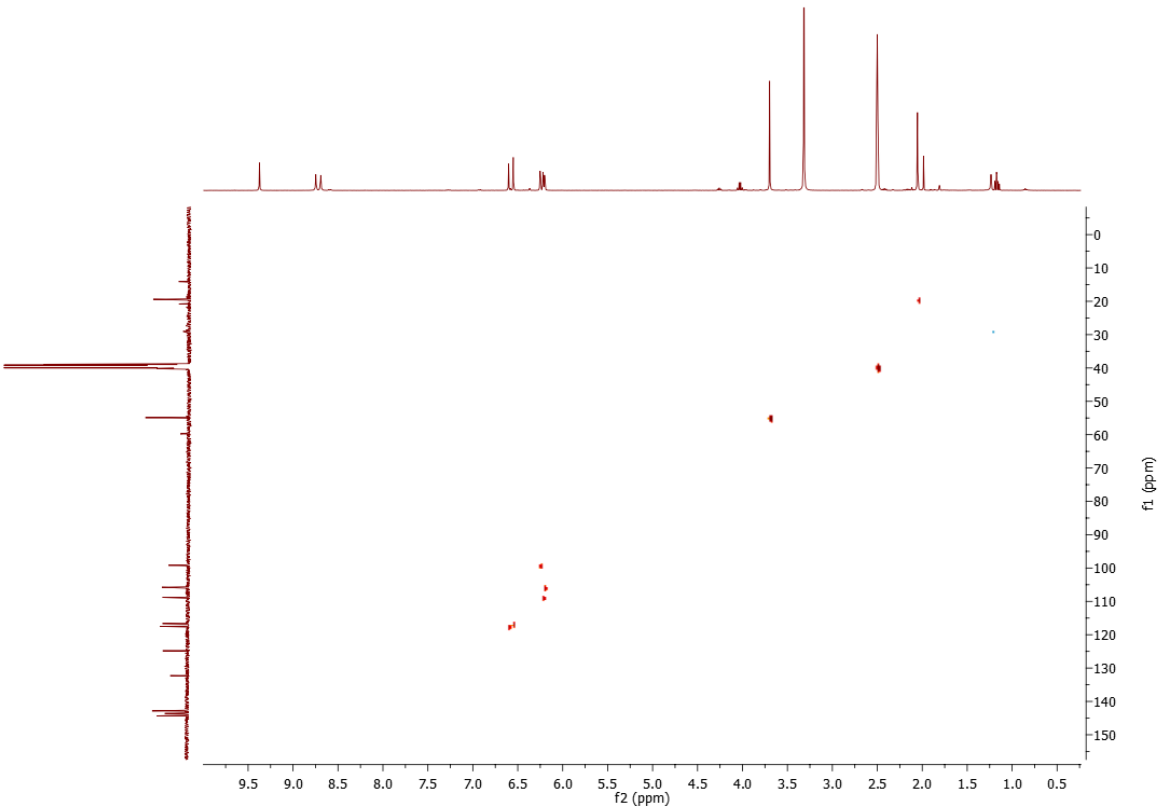


COSY (100 MHz, DMSO-$d_6$)

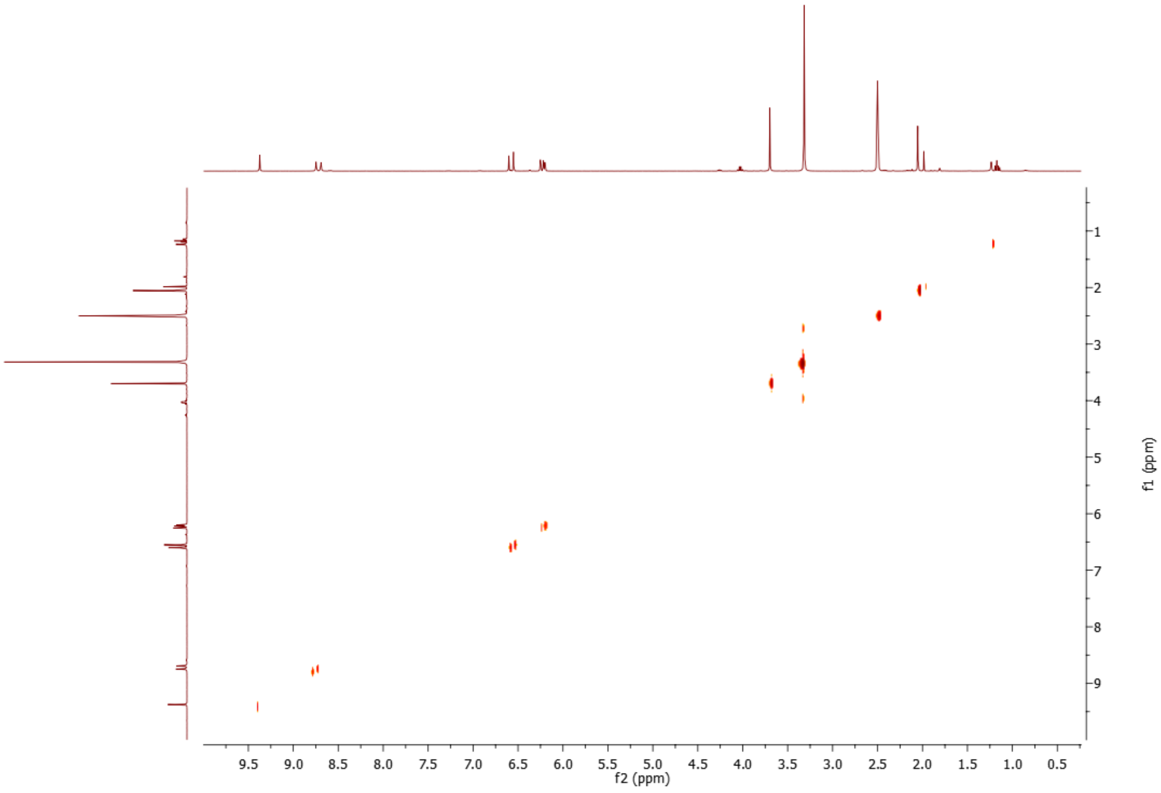

HMBC (100 MHz, DMSO-$d_6$)

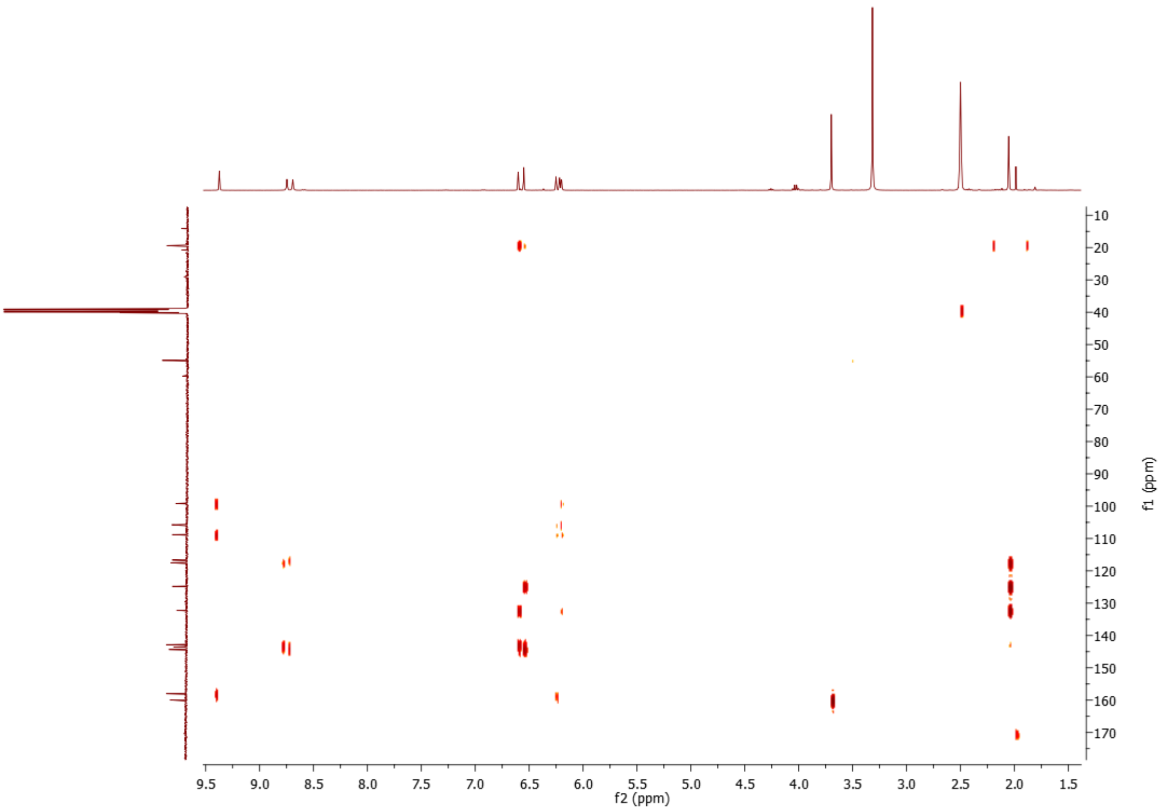

Supplement: File 1 — Experimental procedures and NMR spectra of all new compounds and of decarboxyaltenusin (1). [file Beilstein_J_Org_Chem-17-224-s001.pdf]
